# New insight on the enteric cholinergic innervation of the pig colon by central and peripheral nervous systems: reduction by repeated loperamide administration

**DOI:** 10.3389/fnins.2023.1204233

**Published:** 2023-08-15

**Authors:** Pu-Qing Yuan, Tao Li, Mulugeta Million, Muriel Larauche, Karim Atmani, Jean-Pierre Bellier, Yvette Taché

**Affiliations:** ^1^CURE/Digestive Diseases Research Center, Vatche and Tamar Manoukian Digestive Diseases Division, UCLA David Geffen School of Medicine, Los Angeles, CA, United States; ^2^VA GLAHS, Los Angeles, CA, United States; ^3^Department of Neurology, Brigham and Women’s Hospital, Boston, MA, United States

**Keywords:** central and peripheral cholinergic innervation, choline acetyltransferase, 3D imaging, enteric nervous system, porcine colon

## Abstract

**Introduction:**

The central and peripheral nervous systems provide cholinergic innervation in the colon. The ability to assess their neuroanatomical distinctions is still a challenge. The pig is regarded as a relevant translational model due to the close similarity of its enteric nervous system (ENS) with that of human. Opioid-induced constipation is one of the most common side effects of opioid therapy.

**Methods:**

We developed an approach to differentiate the central and peripheral cholinergic innervation of the pig colon using double immunolabeling with a novel mouse anti-human peripheral type of choline acetyltransferase (hpChAT) antibody combined with a rabbit anti-common type of ChAT (cChAT) antibody, a reliable marker of cholinergic neurons in the central nervous system. We examined their spatial configurations in 3D images of the ENS generated from CLARITY-cleared colonic segments. The density was quantitated computationally using Imaris 9.7. We assessed changes in the distal colon induced by daily oral treatment for 4  weeks with the μ opioid receptor agonist, loperamide (0.4 or 3  mg/kg).

**Results:**

The double labeling showed strong cChAT immunoreactive (ir) fibers in the cervical vagus nerve and neuronal somata and fibers in the ventral horn of the sacral (S2) cord while hpChAT immunoreactivity was visualized only in the ENS but not in the vagus or sacral neural structures indicating the selectivity of these two antibodies. In the colonic myenteric plexus, dense hpChAT-ir neurons and fibers and varicose cChAT-ir fibers surrounding hpChAT-ir neurons were simultaneously visualized in 3D. The density of cChAT-ir varicose fibers in the outer submucosal plexus of both males and females were higher in the transverse and distal colon than in the proximal colon and in the myenteric plexus compared to the outer submucosal plexus and there was no cChAT innervation in the inner submucosal plexus. The density of hpChAT in the ENS showed no segmental or plexus differences in both sexes. Loperamide at the highest dose significantly decreased the density hpChAT-ir fibers + somata in the myenteric plexus of the distal colon.

**Discussion:**

These data showed the distinct density of central cholinergic innervation between myenteric and submucosal plexuses among colonic segments and the localization of cChAT-ir fibers around peripheral hpChAT neurons in 3D. The reduction of cholinergic myenteric innervation by chronic opiate treatment points to target altered prokinetic cholinergic pathway to counteract opiate constipation.

## Highlights

- This study established novel approaches enabling to visualize simultaneously the central and peripheral cholinergic innervation and to quantitate computationally their densities within the enteric nerve system (ENS) of the pig colon.
- The 3D images generated from CLARITY-cleared and immunostained colonic samples revealed that the cholinergic myenteric neurons are closely surrounded by central cholinergic varicose fibers in dot-like structures, nerve terminals.
- Quantitative analysis of the density of colonic cholinergic innervation showed differences between segments and plexuses in the central but not peripheral cholinergic innervation with no sex difference.
- Oral administration of the μ-opioid receptor agonist, loperamide at 3 mg/kg/day for 4 weeks decreased the peripheral cholinergic density in the myenteric plexus of the pig distal colon.

## 1. Introduction

The cholinergic innervation of the colon encompasses the central (extrinsic) and peripheral (enteric) nervous systems (Gonella et al., 1987; Furness et al., 2014; Meerschaert et al., 2022). The central parasympathetic efferent cholinergic nerve fibers originate from neurons in the dorsal motor nucleus of the vagus which innervates more prominently the upper segments of the colon (Berthoud et al., 1991; Browning and Travagli, 2014; Tao et al., 2021). The distal colon receives efferent innervation originating from cholinergic neurons located in the sacral spinal cord with axons running through the pelvic nerve either directly to the colon wall or synapsing onto nerve cell bodies located within the inferior hypogastric plexus with postganglionic fibers entering the colon (Fukai and Fukuda, 1985; Keast and de Groat, 1989; Olsson et al., 2006). However, it is to note that there is inter-species variability to the extent of vagal and pelvic innervation pattern across the length of the colon (Gonella et al., 1987).

The enteric nervous system consists of two main ganglionated layers: the myenteric plexus that lies between the longitudinal and circular layers of the muscularis externa and the submucosal plexuses within the submucosa (Timmermans et al., 1997; Brown and Timmermans, 2004; Furness et al., 2014). In the large mammals, a further division of the submucosal plexuses consists of the outer submucosal plexus adjacent to the luminal side of the circular muscle layer, and the inner submucosal plexus located close to the abluminal side of the lamin muscularis mucosae (Timmermans et al., 2001; Brehmer et al., 2010; Petto et al., 2015). The ENS in the colon is endowed of complex reflex circuits that play a role in controlling several functions including motility, transmucosal fluid exchange, local blood flow and immune status (Furness, 2008; Spencer et al., 2016; Schneider et al., 2019).

Functional and electrophysiological studies indicate that acetylcholine is the primary enteric excitatory neurotransmitter in the gut (Wood, 1987; Furness, 2008). In particular, the administration of exogenous acetylcholine binds to the acetylcholine receptors and promotes colonic mobility *via* the stimulation of fast excitatory synaptic transmission (Schneider and Galligan, 2000; Galligan, 2002). To ascertain precisely the central cholinergic innervation within the different regions of the colon and the spatial configuration of cholinergic central axons and peripheral neurons in the ENS are fundamental to the understanding of the brain-gut interactions *via* cholinergic pathway and to optimize neuromodulation strategies. Of importance is the use of specific markers to discriminate central from peripheral cholinergic nerve fibers and relevant approaches to visualize their three-dimensional (3D) architecture and topography in the colonic ENS. Recently, we produced a novel mouse anti-human peripheral form of choline acetyltransferase (hpChAT) antibody that proved to be without cross-reactivity with the common type of ChAT (cChAT) expressed preferentially in the CNS (Bellier et al., 2019). The hpChAT antibody yielded intense staining in neuronal cells and fibers of the human colon but not in the brain (Bellier et al., 2019). This antibody also allowed us to visualize the peripheral cholinergic innervation of the human sigmoid colon using CLARITY and imaging in 3D structure (Yuan et al., 2021a).

The porcine gastrointestinal (GI) tract is increasingly regarded as a useful research model due to its structural and functional similarities with those of human (Gonzalez et al., 2015). Compared to the mouse, rat, dog, cat or horse, both the pig and human are omnivores and colon fermenters and have similar metabolic and intestinal physiological processes and microbial composition (Kararli, 1995). Among important anatomical similarities relevant to colonic function, there are the taenia and haustra/sacculation which are both present in the pig and human colon, while missing in the dog and most rodents (Miller and Ullrey, 1987; Kararli, 1995; Patterson et al., 2008). In addition, the submucosal plexus encompasses 2–3 layers in the pig and human colon (Hoyle and Burnstock, 1989; Timmermans et al., 2001; Petto et al., 2015) while composed of only one layer in rodents (Kararli, 1995). Additionally, the pig displays a chromosomal structure and genome size with 60% sequence homology and 93% correspondence in relevant biomarkers compared to human (Stevens et al., 1986; Thomas et al., 2003; Murphy et al., 2005). Our recent comparison of transcriptomic profiles in the colonic ENS between the pig and human revealed highly conserved core transcriptional programs with region-dependent patterns between both species (Li et al., 2023). We found that regional-specific transcriptomic responses to vagal nerve stimulation shared 96% of the conserved core transcriptional programs in the myenteric ganglia between the pig and human (Li et al., 2023). Lastly, the size of the pig allows us to use drug dose comparable to that of human as well as to evaluate human-size tailored medical devices (Ziegler et al., 2016). Thus, several components make the pig a preferred translational model (Gonzalez et al., 2015; Lunney et al., 2021).

Opioid drugs are potent pain-relieving medications and prescribed in over 40% of patients including those with advanced cancer (Higginson and Gao, 2012; Gagnon et al., 2015) and other pathologies (Dart et al., 2015). One of the most common side effects of opioid use is constipation (Pappagallo, 2001; Gregorian et al., 2017; Müller-Lissner et al., 2017) that relates to the activation of enteric μ-opiate receptors increasing tonic non-propulsive contractions in the small and large intestine, colonic fluid absorption, and stool desiccation (Bharucha et al., 2013; Mori et al., 2013; Camilleri et al., 2014; Dorn et al., 2014). It has been speculated that a defective cholinergic innervation of human colon may exist in severe chronic idiopathic (slow transit type) constipation (Burleigh, 1988; Bassotti et al., 1993). However, it is still unknown whether repeated opioid administration change the density of the central and peripheral cholinergic innervation of the colon.

In the present study, we first established a new approach to differentiate central and peripheral cholinergic ENS innervation visualized in 3D structures in the pig colon using a modified CLARITY tissue clearing technique combined with the double labeling of cChAT and hpChAT. The tissue clearing technic removed the light scattering lipids from tissue samples, while keeping the proteins and nucleic acids, allowing for deep penetration of macromolecules such as antibodies, and light for fluorescent 3D microscopy (Chung and Deisseroth, 2013; Lai et al., 2016). Secondly, we digitally traced and quantitated the central cholinergic fibers and peripheral cholinergic somata and fibers in the whole mount preparations of ENS using Imaris 9.7 for Neuroscientists, a software for 3D/4D visualization, segmentation and automated tracking of relevant components. Then, we compared the density of central and peripheral cholinergic innervation among the three enteric plexuses and colonic segments in both males and females. Lastly, we examined whether the high-affinity μ-opioid receptor agonist, loperamide (DeHaven-Hudkins et al., 1999), given as a repeated daily oral treatment for 4 weeks alters the density of central and peripheral cholinergic innervation in the pig distal colon.

## 2. Materials and methods

### 2.1. Animals

Eighteen Yucatan minipigs (nine of each castrated males and intact females, ~6 months old, 25–30 kg) were obtained from S&S Farms (Farms, Ramona, CA). They were group housed in pens (either bedding or grate floor, depending on housing availabilities, 2 pigs/pen, 42 ft^2^) in an environmentally controlled room (lights: on/off 6 AM/6 PM, temperature: 61–81°F) under specific pathogen-free conditions. Pigs received *ad libitum* access to diet (5p94 Prolab mini pig diet, PMI Nutrition, St. Louis, MO) and filtered tap water. All animal care and procedures were performed following National Institutes of Health guidelines for the humane use of animals and in accordance with the guidelines of the University of California at Los Angeles (UCLA) Institutional Animal Care and Use Committee, Chancellor’s Animal Research Committee (ARC) (approval protocol 2018-074-01). All efforts were made to avoid suffering.

### 2.2. Treatments

Six pigs (three of each sex) were left untreated (naïve pigs). The remaining twelve pigs were randomly divided into three groups (4/group, 2 of each sex) and given orally 0.4 or 3 mg/kg/day loperamide hydrochloride (2 mg/caplet, SDA Laboratories, Wellspring Meds, USA) for four weeks. The caplets were embedded in a palatant containing bananas, marshmallows or yogurts mixed with honey. The vehicle group received oral palatant only.

### 2.3. Tissue collections

Naïve pigs were fasted for 12 h and anaesthetized by an intramuscular injection of midazolam (1 mg/kg, cat. # 067595, Covetrus, Dublin, OH), ketamine (15 mg/kg, cat. # 068317, Covetrus) and meloxicam (0.3 mg/kg, cat. # 049755, Covetrus). This was followed with an intravenous injection of pentobarbital (100 mg/kg, Abbott Laboratories, North Chicago, IL) for euthanasia followed by collection of colonic tissues. It is to note that the longitudinal muscle layer of the pig proximal colon is thickened into three muscular bands named the taeniae, and the wall between the taeniae bulges out to form rows of sacculation, the haustra. The centripetal part of the pig proximal colon has two taeniae and two rows of haustra, which gradually disappear in the centrifugal part (Hedemann et al., 2002). The full thickness colonic samples (about 3–4 cm long) were harvested between the taenia about 10 cm from the ceco-colic junction for the proximal colon, about 10 cm from the end of the proximal segment for the transverse colon (about 10 cm from the end of the centrifugal spiral colon) and about 20 cm from the ano-rectum for the distal colon. The cervical vagal nerve (left side, 1 cm) and sacral spinal cord (S2, 1 cm) were dissected from one male naïve pig. In opiate-treated pigs, the colonic specimens were collected from the distal colon 12 h after the last oral administration of vehicle or loperamide as described above. All samples were immediately immersed in Belzer UW^®^ Cold Storage Solution (Bridge to Life Ltd., Columbia, SC) on ice.

### 2.4. Clarity clearing of the muscularis externa

The protocol of passive CLARITY for human colon detailed previously (Yuan et al., 2021a) was modified to clear the muscularis externa of pig proximal colon collected between the taeniae where the layer of longitudinal muscle is thinner and easier to perform the CLARITY protocol and whole mount preparation. Briefly, two pieces of proximal colon (3 × 4 cm, one naïve male pig) were pinned-flat with mucosa facing up on the Sylgard-coated dishes and immersed in ice-cold 4% paraformaldehyde (Sigma-Aldrich, St Louis, MO) in phosphate buffered saline (PBS). On the second day, the mucosa, submucosa and serosa were peeled off from flat samples and the muscularis externa was cut into small pieces (1 × 1 cm) and then immersed in an ice-cold hydrogel solution containing a mixture of 4% acrylamide (BIO-RAD, Hercules, CA) and 0.25% VA044 (Wako, Richmond, VA) in 1× PBS (pH 7.4) and maintained at 4°C for 3 days for hydrogel-tissue hybridization. Samples were transferred to 50 mL conical tubes containing 15 mL of fresh hydrogel solution to replace all oxygen (known to inhibit the acrylamide polymerization) with pure nitrogen gas. The hydrogel polymerization was initiated by submerging the conical tubes with tissue samples in a temperature-controlled 37°C water bath (Precision Scientific Water Bath Model 182, Thermo Scientific, Marietta, Ohio) for 3 h. The excess hydrogel monomers were washed out from inside the muscularis externa on a shaker/rotator plate (70 rpm, New Brunswick Scientific Co., Edison, NJ) with a clearing solution containing 4% sodium dodecyl sulfate (Sigma-Aldrich) in sodium borate buffer (200 mM, pH 8.5) (Sigma-Aldrich) at 37°C for about 1 week until clearing is achieved. Thereafter, samples were placed in 0.1% Triton-X 100 (Sigma-Aldrich) in 1× PBS (pH 7.4) on a shaker plate (New Brunswick Scientific Co.) for one day (37°C, 70 rpm) to wash out sodium dodecyl sulfate micelles. A previous report indicated that omitting paraformaldehyde and bisacrylamide from the original hydrogel composition including 4% paraformaldehyde, 4% acrylamide, 0.05% bis-acrylamide and 0.25% VA-044 did not change the degree of protein loss compared to paraformaldehyde-fixed tissue in 8% sodium dodecyl sulfate clearing bath solutions (Chung and Deisseroth, 2013). This protocol speeded up CLARITY tissue clearing and made the tissue more porous, facilitating antibody tissue penetration and improving immunolabeling (Yang et al., 2014). The immunostaining performed on samples prepared with this modified protocol showed the same intensity as cleared tissue samples processed with the original hydrogel solution (Yang et al., 2014). We observed that tissue samples prepared with 4% acrylamide and 0.25% VA-044 exhibited slightly more swelling than those processed with the original hydrogel solution. However, they shrank back to their original size within a few hours in the 0.1% Triton-X 100 PBS as previously reported (Yang et al., 2014). We did not find perceptible alterations in the tissue structure and morphology caused by the tissue volume expansion-contraction.

### 2.5. Whole mount preparation of colonic enteric plexuses

Colonic samples (2 × 4 cm) dissected in the area between the taeniae from the proximal, transverse and distal colon of naïve pigs (*n* = 6, 3 of each sex) and distal colon of vehicle- or loperamide-treated pigs (*n* = 12, 6 of each sex) were stretched in both dimensions, pinned-flat with mucosa facing up on the Sylgard-coated dishes and immersed in an ice-cold 4% paraformaldehyde in PBS overnight at 4°C. After washing with PBS at room temperature for 3 × 30 min, the mucosa layer was scraped off. The whole submucosa was peeled off from the muscular externa and separated into the inner submucosal and outer submucosal plexuses with a curved fine spring scissors (No. 15123, Fine Science Tools, Foster City, CA). The circular muscle was removed bundle by bundle and then the preparations were turned over to remove fat and blood vessels from the serosa. The myenteric plexus adhered to the longitudinal muscle.

### 2.6. Cryostat sectioning of cervical vagal nerve and sacral spinal cord

The dissected cervical vagus nerve and sacral spinal cord at S2 were fixed overnight in 4% paraformaldehyde in PBS at 4°C, cryoprotected overnight in 20% sucrose in PBS and subsequently embedded in optimal cutting temperature compound (OCT, Thermo Fisher Scientific, Waltham, MA) on dry ice. Samples were cut in 25-μm thick coronal sections with a cryostat (Leica CM3050 S, Leica Microsystems Nussloch GmbH, Nussloch, Germany) at -20°C.

### 2.7. Double immunostaining

CLARITY-cleared samples, whole mount preparations and frozen sections were transferred into Corning™ Costar™ Flat Bottom 24 Well Plates (Fisher Scientific, Hampton, NH) for free-floating double immunolabeling with the samples floating in solution for antibody incubation and washing under gentle shaking (90 rpm, Corning™ LSE™ Low Speed Orbital Shaker, Fisher Scientific, Canoga Park, CA). After immersion in 10% normal donkey serum in PBS with 0.3% Triton-X 100 (one day for CLARITY-cleared samples at 4°C, 30 min for whole mount preparations and frozen sections at room temperature) to block non-specific interactions and enhance permeabilization, samples were incubated with primary antibodies followed by fluorescent-dye conjugated secondary antibodies (Table 1). Samples were not mounted on slides until after completing the immunohistochemistry process.

**Table 1:** Immunofluorescence reagents.

	Name	Immunogen	Host	Source/product no.	Dilution
Primary antibodies	hpChAT	CSYKALLDRTQSSRK (Pat. 04412018JP, 2018)	Mouse	Dr. Bellier/H3	1:2,000–4,000
	cChAT	FVLDVVINFRRLSEGDLFTQLRKIVKMASNEDERLPPIGLLTSDGRSEWAKARTVLLKDSTNRDSLDMIERCICLVCLDGPGTGELSDTHRALQLLHGGGCGLNGANRWYDKSLQ, plus MRGSHHHHHHGS of N-terminal Tag and PSLIS of 3′ region	Rabbit	Dr. Kimura/98	1:1,000
	ChAT	human placental ChAT	Goat	Chemicon/AB144p	1:200
	HuC/D	human HuC/HuD neuronal protein	Mouse, monoclonal (clone 16A11)	Life Technologies/A-21271	1:500
Secondary antibodies	Alexa 405-conjugated anti-mouse IgG	mouse IgG (H + L)	Donkey	Abcam/ab175659	1:400
	Alexa 488-conjugated anti-rabbit IgG	rabbit IgG (H + L)	Donkey	Jackson Imm Res/AB_2340846	1:400
	Alexa 555-conjugated anti-goat IgG	goat IgG (H + L)	Donkey	Abcam/ab150062	1:400
Normal serum	Normal donkey serum	(−)	Donkey	Jackson Imm Res	1:10

#### 2.7.1. hpChAT and HuC/D

To determine the capacity of the novel mouse anti-hpChAT antiserum (Bellier et al., 2019) to label pig colonic enteric neurons, a double immunolabeling of hpChAT and HuC/D, a pan-neuronal marker (Lin et al., 2002) was performed with whole mount preparations of myenteric plexus from one naïve pig using a sequential staining procedure which allowed us to use two mouse monoclonal antibodies in the same preparation (Ho et al., 2003). The preparations were incubated first with the mouse anti-hpChAT antibody for 5 days at 4°C followed by donkey anti-mouse Alexa Fluor^®^ 488 (Table 1) for 3 h at room temperature. Samples were then incubated with mouse anti-HuC/D for 1 day at 4°C followed by donkey anti-mouse Alexa Fluor^®^ 405 for 3 h at room temperature. This strategy allows us to determine the feasibility of this sequential staining by visualizing whether we can detect neurons only labeled by HuC/D (blue color). Samples were washed with PBS at room temperature for 1 h after each first and second antibody incubation and then were mounted onto Fisherbrand™ Superfrost™ Plus Microscope Slides (Fisher Scientific, Hampton, NH) with VECTASHIELD^®^ antifade mounting medium (Vector Laboratories, Newark, CA). The double labeling of hpChAT and HuC/D were also performed with the whole mount preparations of myenteric plexus from the distal colon of vehicle- or loperamide-treated pigs to determine the alterations of total and cholinergic myenteric neurons.

#### 2.7.2. hpChAT and ChAT (AB144p)

To verify the selectivity of hpChAT to label pig enteric cholinergic neurons and fibers, a double labeling was performed with hpChAT and a goat anti-human ChAT antibody (ChAT AB144p, Chemicon International, Temecula, CA). The latter is commonly used to label enteric cholinergic neurons (Hao et al., 2013). The whole mount preparations of myenteric plexus from one naïve male pig were incubated with a mixture of two first antibodies for 5 days at 4°C followed by a mixture of secondary donkey anti-mouse Alexa Fluor^®^ 488 and donkey anti-goat Alexa Fluor^®^ 555 (Table 1) for 1 day at room temperature. Other steps were the same as described above.

#### 2.7.3. hpChAT and cChAT

To distinguish and visualize simultaneously central and peripheral cholinergic innervation in the pig colon, double immunostaining with mouse antibody anti-hpChAT and a rabbit antibody against rat cChAT (Kimura et al., 2007) was performed with three preparations: (i) CLARITY-cleared colonic muscular externa samples for 3D imaging, (ii) whole mount preparations for quantitative analysis and (iii) cryostat sections of cervical vagal nerve and sacral spinal cord to confirm the selectivity of hpChAT and cChAT antibodies. The procedures were the same as described above except that ChAT144p antibody and donkey anti-goat Alexa Fluor^®^ 555 were replaced by cChAT antibody and donkey anti-rabbit Alexa Fluor^®^ 555 (Table 1). After double immunostaining, CLARITY-cleared samples were immersed for one day in a custom-made refractive index matching solution containing 88% Histodenz (Sigma-Aldrich) in 0.02 M phosphate buffer with 0.01% sodium azide (Sigma-Aldrich) (pH 7.5) with a refractive index of 1.46 at 4°C. Then, samples were mounted with fresh refractive index matching solution in a sealed watertight well prepared with iSpacers (SunJin Lab, Hsinchu City, Taiwan) which are made from different thickness adhesive tapes. Whole mount preparations and sections were mounted onto Fisherbrand™ Superfrost™ Plus Microscope Slides (Fisher Scientific, Hampton, NH) with VECTASHIELD^®^ antifade mounting medium (Vector Laboratories, Newark, CA). Negative control samples were also subjected to the same procedures without primary antibodies.

### 2.8. 3D imaging/digital tracing and quantification

Z-stack images were acquired from CLARITY-cleared samples and whole mount preparations with Zeiss LSM 710 confocal microscope (Carl Zeiss Microscopy, LLC, White Plains, NY). The Alexa Fluor^®^ 405, 488 and 555 were excited using a 405 nm violet laser, 488 nm Argon laser and a 561–10 nm diode-pumped solid-state laser, respectively. The Z-axis intervals were 1 μm with depths 35 μm for the myenteric plexus in CLARITY-cleared samples, and 20 μm for myenteric plexus and 45 μm for inner and outer submucosal plexuses for whole mount preparations. The confocal parameters were determined, and the same setting was applied for each sample. The Z-stack images were collected using Zen image collection software (Carl Zeiss Microscopy) and processed for 3D reconstruction using Imaris 9.7 for Neuroscientists (Bitplane Inc., Concord, MA). The traditional procedure of counting nerve fibers manually *via* grid-based stereology and histomorphometry is both time consuming and labor intensive (Hunter et al., 2020). We adapted the Imaris 9.7 Surfaces Rendering Technology^1^ and developed a protocol for the density measurement of cholinergic innervation in 3D Images of the pig colon.^2^ Briefly, the surfaces of cChAT immunoreactive (ir) nerve fibers, hpChAT-ir fibers + somata and ganglia containing neurons and fibers were created automatically with the ImarisSurface function. The setting of the parameters for ImarisSurface were surface grain size: 1.38 μm, upper background subtraction threshold values: 1.28 μm for green channel (hpChAT) and 1.42 μm for red channel (cChAT), lower background subtraction threshold values: 0.96 μm for green channel (hpChAT) and 1.10 μm for red channel (cChAT). The volumes of these marked surfaces were automatically measured using the software Imaris 9.7. The densities of cChAT-ir nerve fibers or hpChAT-ir fibers plus somata were calculated and expressed as percentage of their volumes in ganglion volumes (v/v, %). HuC/D-ir myenteric neurons in the distal colon of pigs treated with loperamide or vehicle were counted automatically with ImarisSpot function in 6 ganglia/3D image. The density of HuC/D-ir neurons was calculated and expressed as the numbers per ganglion volumes (number/mm^3^). The 5–6 3D images generated from each plexus in the three colon segments were used for the quantitative analysis. This approach allowed us to perform direct, objective and automatic measurements with less biases due to observer/examiner judgment. It was also faster than counting manually and enabled to quantify a large number of samples increasing the statistical accuracy. The quantitation in the distal colon of loperamide experiment was performed in a blind manner and the information on treatments (vehicle, low or high dose of loperamide) was withheld until after the quantitation was completed.

For the double labeling of hpChAT and HuC/D or hpChAT and ChAT (AB144p) with two pieces of whole mount preparations of proximal colonic myenteric plexus from one naïve pig, neurons were manually counted in 5 ganglia of each preparation and the values were pooled together. The percentages of co-labeled neurons by hpChAT and HuC/D or hpChAT and ChAT (AB144p) in total HuC/D-ir or ChAT (AB144p)-ir neurons were calculated.

### 2.9. Statistical analysis

The statical analysis of the cholinergic innervation density between colonic segments and plexuses and HuC/D positive myenteric neurons in distal colonic segment of vehicle or loperamide treated pigs were performed with one-way analysis of variance (ANOVA) followed by Tukey’s *post hoc* test. Sex differences were assessed by *t*- test. A *p* value <0.05 was considered statistically significant.

## 3. Results

### 3.1. hpChAT-ir neurons are co-labeled by anti- HuC/D or ChAT antibody (AB144p) in the myenteric plexus of proximal colon in a naïve pig

**Figure 1:**
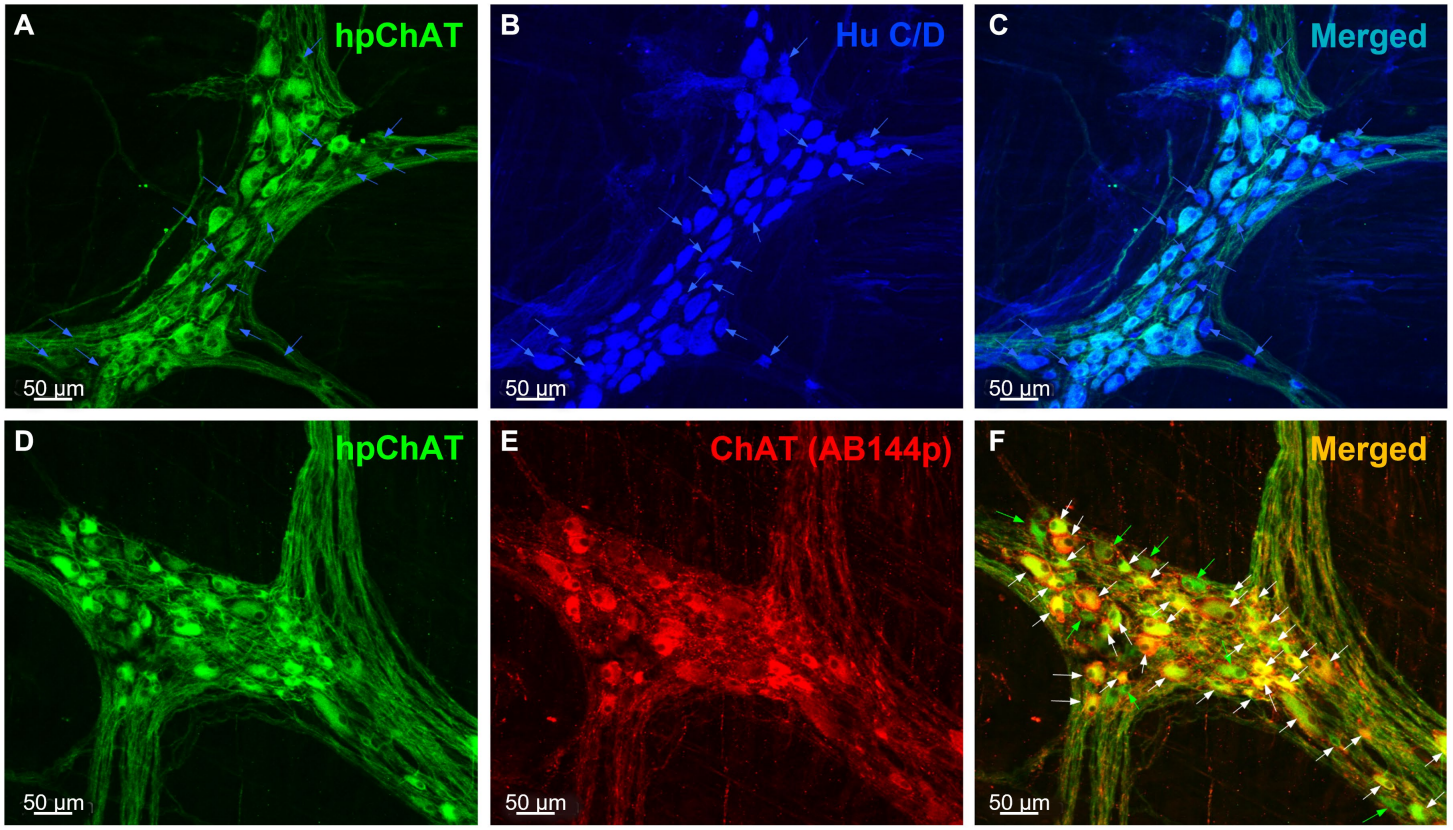
hpChAT immunoreactive cells were co-labeled by HuC/D and ChAT (AB144p) in the myenteric plexus of proximal colon in a naïve pig. Double immunostaining using a sequential staining procedure with antibodies against human peripheral choline acetyltransferase [hpChAT, green, **(A,D)**] and HuC/D, a marker for pan-neurons [blue, **(B)**] or ChAT (AB144p) [red, **(E)**], a well-recognized marker for cholinergic neurons showed the cholinergic neurons and fibers in the whole-mount preparations of the longitudinal muscle-myenteric plexus in the proximal colon of a naïve male pig. **(C,F)** Merged images of **A** and **B**, **D** and **E**. In the upper panel, the blue arrows point to neurons labeled by HuC/D only. In the lower panel, white or green arrows point to neurons double-labeled by hpChAT and ChAT AB144p or single-labeled by hpChAT, respectively. Bar scale in each panel 50 μm.

The double immunolabeling of hpChAT (Figures 1A,D) and HuC/D (Figure 1B) in the myenteric plexus of the proximal colon using a sequential staining procedure showed that hpChAT-ir cells were fully co-labeled by HuC/D antibody, a marker used for the evaluation of neuronal populations. This demonstrates that cells labeled by hpChAT in the myenteric plexus are neurons (Figure 1C). Of HuC/D positive cells, 71% were hpChAT-ir while 29% were only labeled by HuC/D, indicating the feasibility of the sequential staining in the same preparation with hpChAT and HuC/D antibodies both raised from the mouse. The hpChAT immunostaining was visualized in the neuronal cell bodies and processes forming bundles separating the ganglia (Figures 1A,C). By contrast, the anti-HuC/D antibody uniformly labeled the cytoplasm and often also the nucleus of all neuronal cell bodies but not the neuronal processes (Figures 1B,C). No immunoreactivity was observed in preparations incubated with the antibody diluent alone instead of primary antibodies (data not shown).

The double labeling with the mouse anti-hpChAT antiserum (Figure 1D) and the goat anti-ChAT antibody (AB144p) (Figure 1E), a ChAT antibody commonly used to label enteric cholinergic neurons, showed that the immunoreactivity of hpChAT mostly overlapped (83%) with that of ChAT in the myenteric plexus of pig proximal colon (Figure 1F). Compared to ChAT (AB144p) (Figure 1E), hpChAT immunostaining (Figures 1A,D) provided a more intense, clearer and sharper neuronal profile, with more detailed structures and fine fibers with high resolution. No immunoreactivity was observed in the preparations incubated with the antibody diluent alone instead of primary antibodies (data not shown).

### 3.2. The central and peripheral cholinergic nerve fibers and neurons were distinctively labeled by cChAT and hpChAT, respectively, in the pig colon

**Figure 2:**
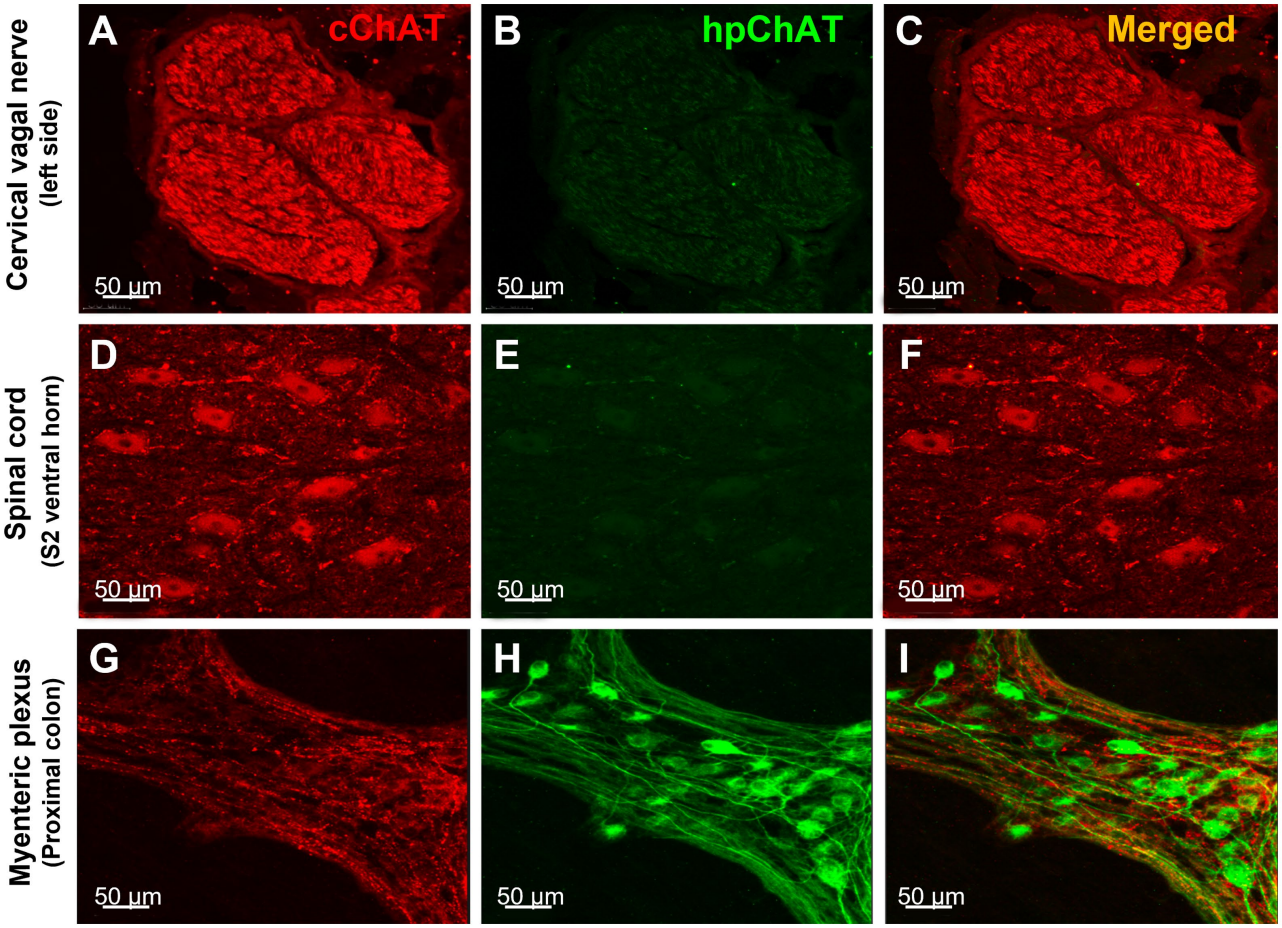
hpChAT antibody only labeled the cholinergic nerve fibers and neurons in the colonic myenteric plexus while cChAT antibody labeled the cervical vagal nerves and sacral spinal cord neurons in a naïve pig. Double immunostaining with antibodies against common type of choline acetyltransferase [cChAT, red, **(A,D,G)**] expressed preferentially in the central nervous system, and human peripheral choline acetyltransferase [hpChAT, green, **(H,I)**] showed the cChAT labeling of cholinergic neurons/fibers in the cervical vagal bundles **(A,C)**, spinal cord sacral S2 ventral horn **(D,F)** and in fibers of the whole-mount preparations of the longitudinal muscle-myenteric plexus of the proximal colon **(G)** while the hpChAT labeling only in the neurons and fibers in myenteric plexus **(H,I)** and not in the vagus or spinal cord **(B,E)** of a naïve male pig. **(C,F,I)** Merged images of **A** and **B**, **D** and **E**, and **G** and **H**, respectively. No overlapping of cChAT and hpChAT was found in **I**. Bar scale in each panel 50 μm.

The double labeling with a rabbit antibody against cChAT and the mouse antibody against hpChAT showed strong cChAT immunoreactivity in fibers of the pig cervical vagus nerve and no labeling in the perineurium (Figures 2A,C). The cChAT labeling was also observed in neuronal somata and fibers of the ventral horn in the pig sacral (S2) spinal cord (Figures 2D,F) while there was no hpChAT immunoreactivity visualized in these structures (Figures 2B,E). By contrast, in the pig proximal colonic myenteric plexus, intense hpChAT immunoreactivity was displayed in both neuronal somata and fibers (Figure 2H) while cChAT immunoreactivity was visualized in the fibers with varicose morphology but not in somata (Figure 2G). There were rare cChAT-ir fibers co-labeled with hpChAT-ir (Figure 2I).

### 3.3. 3D images displayed spatial configuration of central and peripheral cholinergic innervation in the pig colonic myenteric plexus

**Figure 3:**
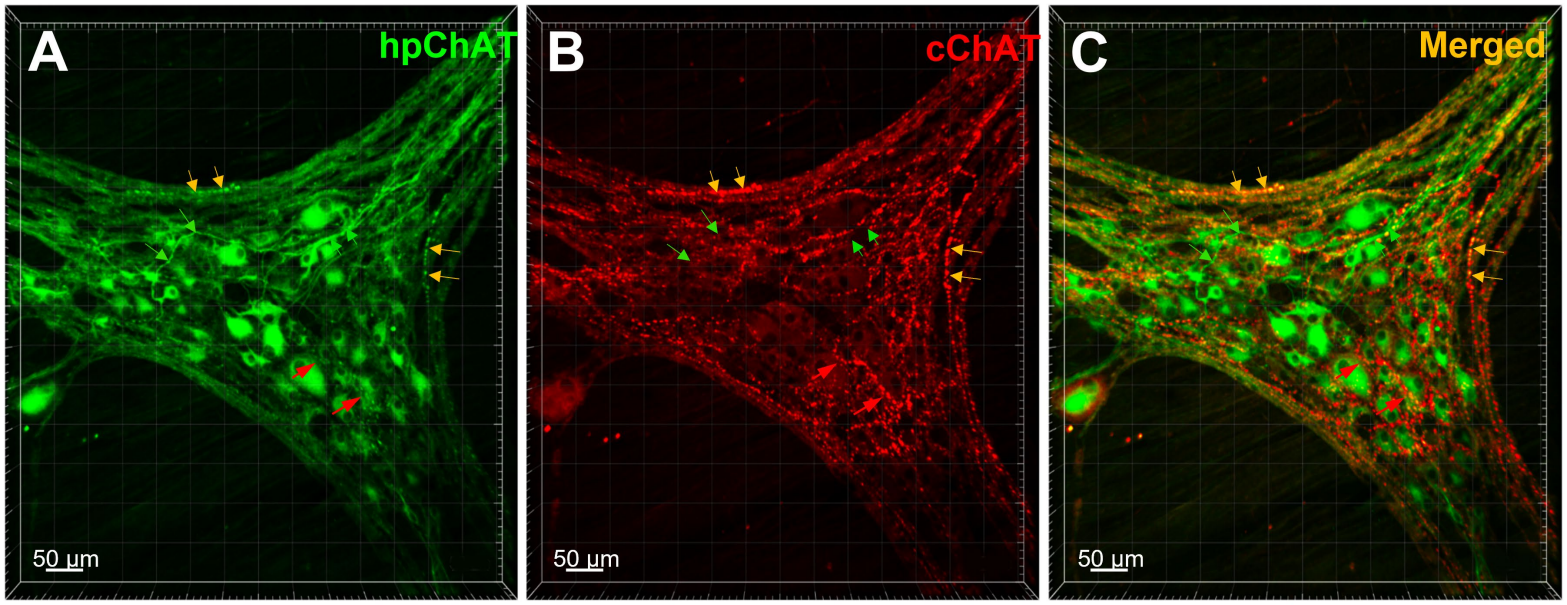
The central and peripheral cholinergic nerve fibers and neurons were distinctly labeled by cChAT and hpChAT in the pig colonic myenteric plexus. Double immunostaining with antibodies against human peripheral form of choline acetyltransferase [hpChAT, green, **(A)**] and a common type of choline acetyltransferase expressed preferentially in the central nervous system [cChAT, red, **(B)**] showed central and peripheral cholinergic neurons/fibers in the whole-mount preparations of the longitudinal muscle-myenteric plexus in the porcine proximal colon. **(C)** Merged image of **A** and **B**. The green arrows point cholinergic fibers labeled by hpChAT antibody in **A, C** while not labeled by cChAT in **B**. The red arrows point to central cholinergic fibers with varicose morphology labeled with cChAT in **B, C** but not by hpChAT in **A**. Yellow arrows point to the fibers double-labeled by both hpChAT and cChAT which are thought to be peripheral myenteric due to the specificity of hpChAT for labeling exclusively peripheral cholinergic neurons/fibers. Bar scale in each panel 50 μm.

**Figure 4:**
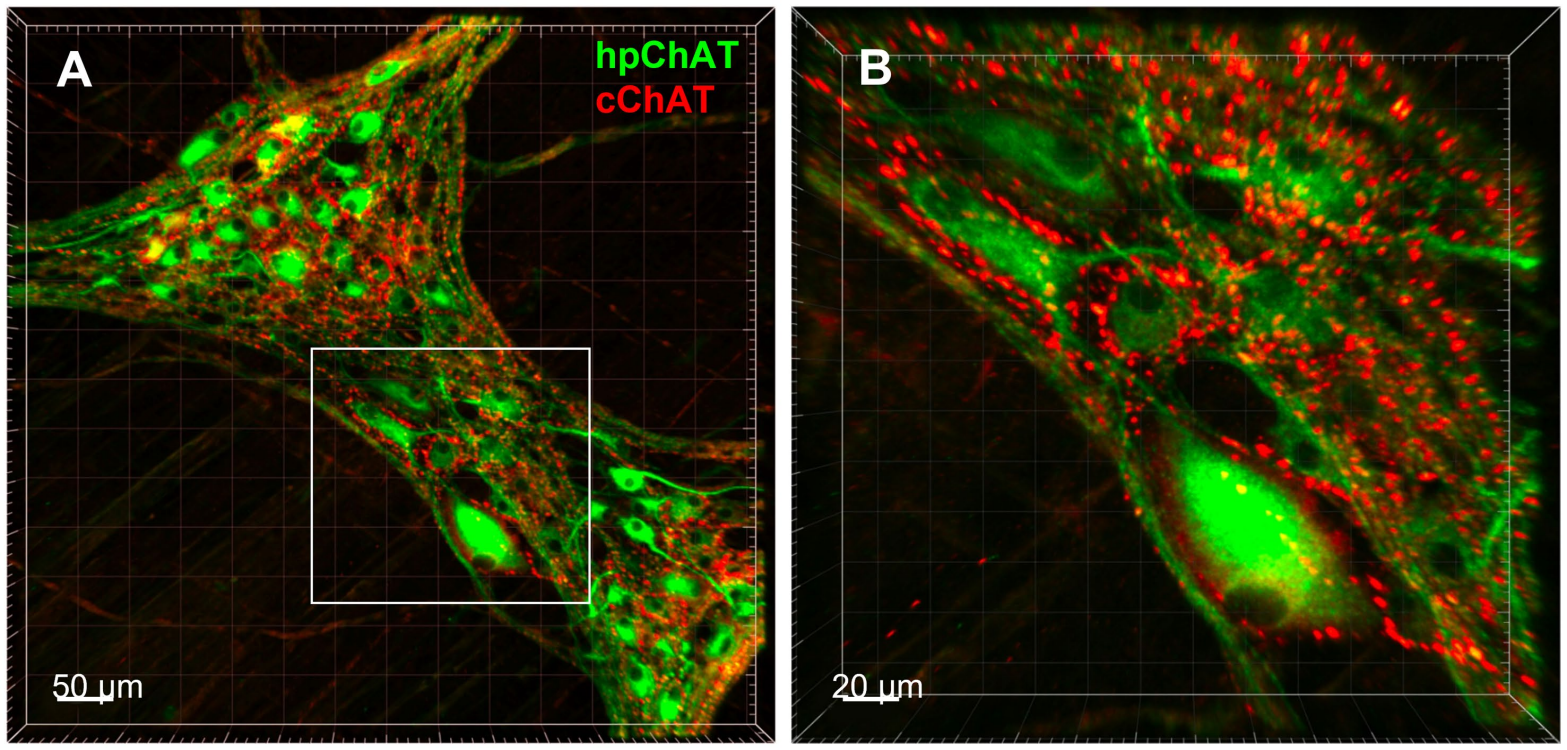
3D Images displayed spatial configuration of central and peripheral cholinergic innervation in the pig colonic myenteric plexus. 3D images demonstrated the spatial configuration of central and peripheral cholinergic innervation in the myenteric plexus in the porcine proximal colonic muscularis externa generated with a modified CLARITY tissue clearing protocol developed in this study. **(A)** Peripheral cholinergic neurons and fibers were labeled with human peripheral choline acetyltransferase (hpChAT) antibody as green color, while central cholinergic innervation was labeled with common type of ChAT (cChAT) antibody as red color. Bar scale 50 μm. **(B)** High magnification of the insert in A showing that hpChAT immunoreactive (ir) neurons are surrounded by cChAT-ir varicose fibers and dot like structures, presumably nerve terminals. Bar scale 20 μm.

Using a modified CLARITY protocol developed for pig colon, we obtained a transparent hydrogel-muscularis externa hybridization without light-scattering lipids which allowed us to image deeply and acquire high quality confocal Z-stack images of myenteric plexus for 3D reconstruction. The 3D images exhibited a clear spatial view of the cholinergic nerve network in the myenteric plexus double labeled with hpChAT and cChAT antibodies. The peripheral cholinergic neurons (hpChAT-ir) were heterogeneous with multi-dendritic, bipolar or uni-axonal morphology and distributed in a single layer located between the longitudinal and circular muscle layers (Figure 3A). Each hpChAT-ir neuron sent a single axon and proceeded into the strands (Figure 3A). The central cholinergic fibers (cChAT-ir) were varicose and cChAT-ir neurons were rarely observed (Figure 3B). In the merged images, most nerve fibers were separately labeled by hpChAT and cChAT and only few co-labeled fibers were occasionally observed (Figure 3C). 3D images with highest magnifications showed that myenteric cholinergic neuronal cell bodies were closely surrounded by central cholinergic varicose fibers with dot-like structures (Figures 4A,B).

### 3.4. Quantitative analysis showed distinct densities of central and peripheral cholinergic innervation within three enteric plexuses along the different segments of the pig colon

**Figure 5:**
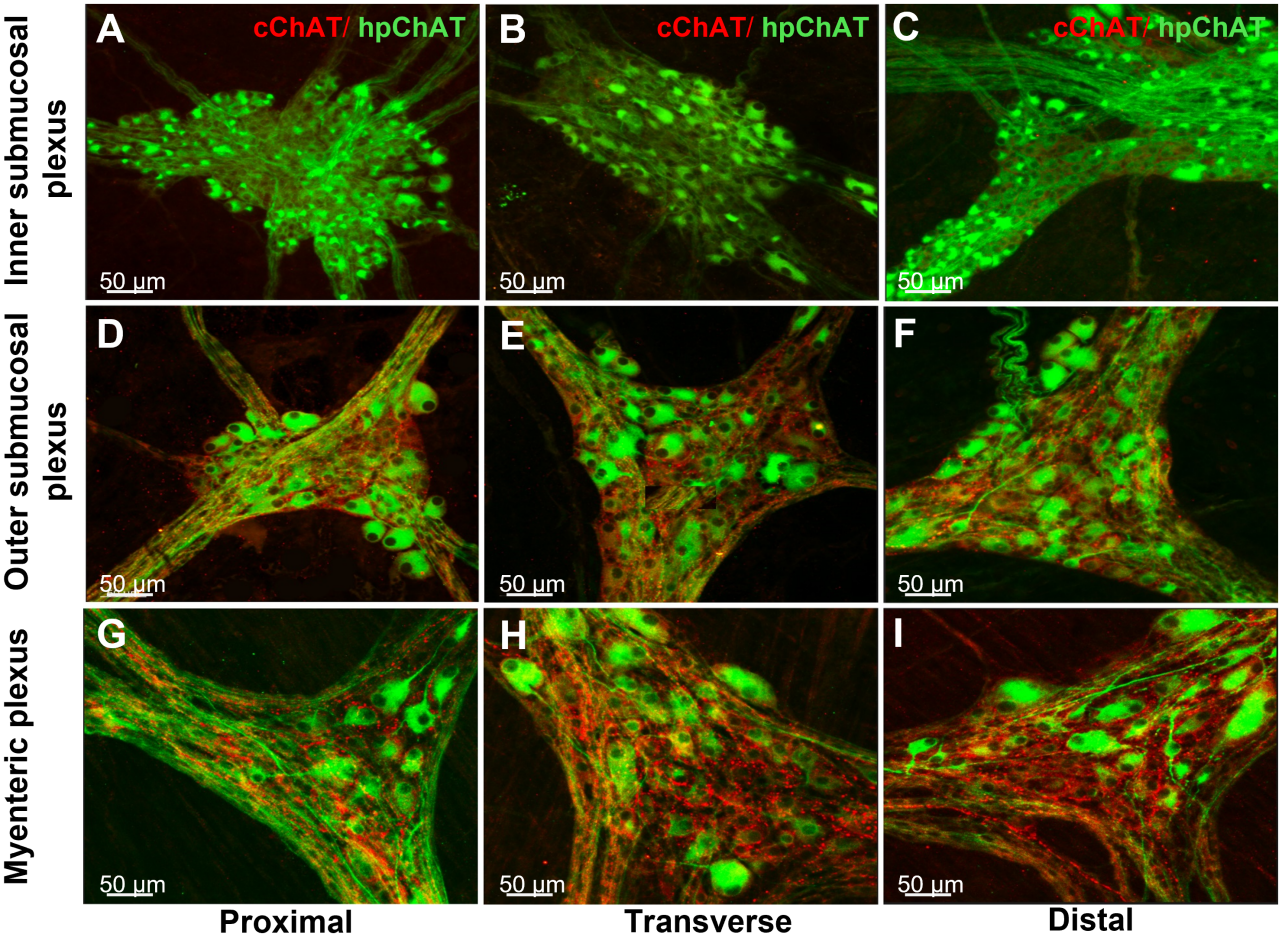
Regional differences of central and peripheral cholinergic innervation in the enteric nerve system of the pig colon. Double labeling of central and peripheral cholinergic innervation in the inner **(A–C)** and outer submucosal plexus **(D–F)** and myenteric plexus **(G–H)** of the porcine proximal **(A,D,G)**, transverse **(B,E,H)** and distal colon **(C,F,I)** colon. Peripheral cholinergic neurons and fibers were labeled with hpChAT antibody as green color, while central cholinergic innervation was labeled with cChAT as red color. cChAT immunoreactive (ir) varicose fibers and dot like structures surround the hpChAT-ir neurons. cChAT-ir fibers were visualized in the outer submucosal and myenteric plexus where it was denser in the transverse and distal colon than in the proximal colon while there was no cChAT-ir fibers in the inner submucosal plexus. Similar density of hpChAT-ir neurons/fibers was displayed among three segments within each plexus. Bar scale in each panel 50 μm.

The 3D images generated from the whole mount preparations of the proximal, transverse and distal colon clearly showed that the inner submucosal plexus had no cChAT-ir fibers (Figures 5A–C), while the outer submucosal plexus contains labeled fibers (Figures 5D–F) and the myenteric plexus displayed varicose cChAT-ir fibers that were denser in the transverse (Figure 5H) and distal (Figure 5I) than proximal colon (Figure 5G). By contrast, hpChAT immunostaining showed similar density among the three segments and in each plexus (Figures 5A–I). However, hpChAT-ir neurons displayed remarkable differences in the morphology (size, shape, processes) between the three plexuses. In the inner submucosal plexus, neurons in each ganglion are of a small size, uniform shape and dense (Figures 5A–C). In the outer submucosal plexus, hpChAT-ir neurons exhibited various sizes and shapes and a less dense distribution (Figures 5D–F) than those in the inner submucosal plexus (Figures 5A–C). In the myenteric plexus, hpChAT-ir neurons were more heterogeneous (Figures 5G–I) than in the outer submucosal plexus. In addition, a prominent difference was observed in the density and size of ganglia labeled by hpChAT among the three plexuses with small size but dense neuronal population in the inner submucosal plexus, less dense with a wider meshwork in the outer submucosal plexus and large size in the myenteric plexus under the low magnification (Figure 5).

**Figure 6:**
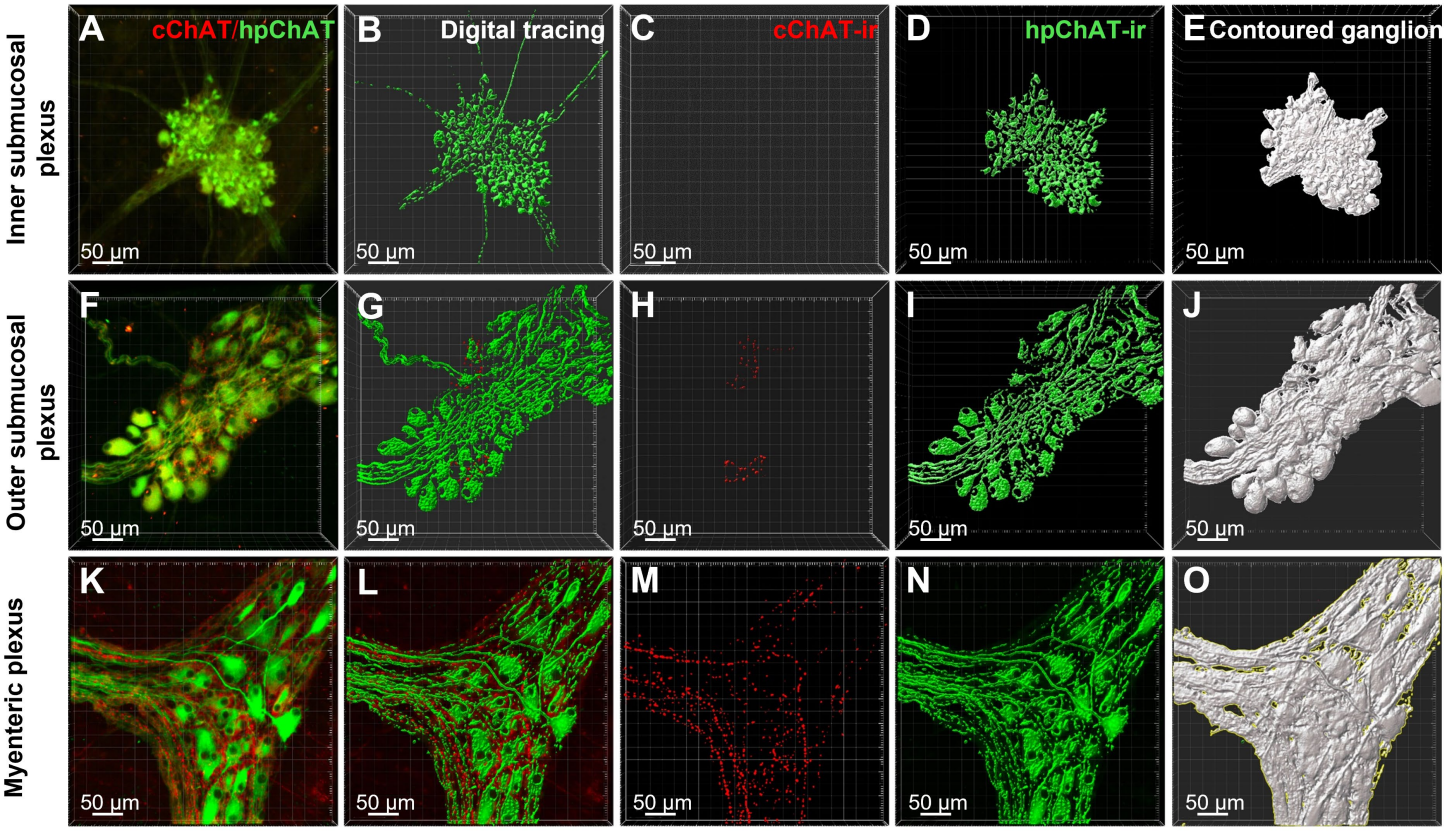
cChAT immunoreactive (ir) fibers and hpChAT-ir fibers + somata were digitally traced with ImarisSurface function and the ganglia containing cholinergic neurons/fibers were delimited for computational quantitation with Imaris 9.7. 3D images with the volume X × Y × Z (axis) = 708 × 708 × 20–45 (μm^3^) were acquired from the whole-mount preparations of inner submucosal plexus, outer submucosal plexus and myenteric plexus in the porcine proximal colon. cChAT-ir fibers and hpChAT-ir fibers + somata in the inner submucosal plexus **(A)**, outer submucosal plexus **(F)** and myenteric plexus **(K)** were digitally traced with Imaris SurfaceFunction **(B,G,L)** respectively and extracted separately **(C,H,M)** and **(D,I,N)**. The ganglia containing cholinergic neurons/fibers were demarcated **(E,J,O)**. The volumes of traced nerve fibers and neurons and outlined ganglia were automatically measured with Imaris 9.7 for quantitation of densities of positive cChAT fibers and hpChAT fibers + somata. Bar scale in each panel 50 μm.

**Figure 7:**
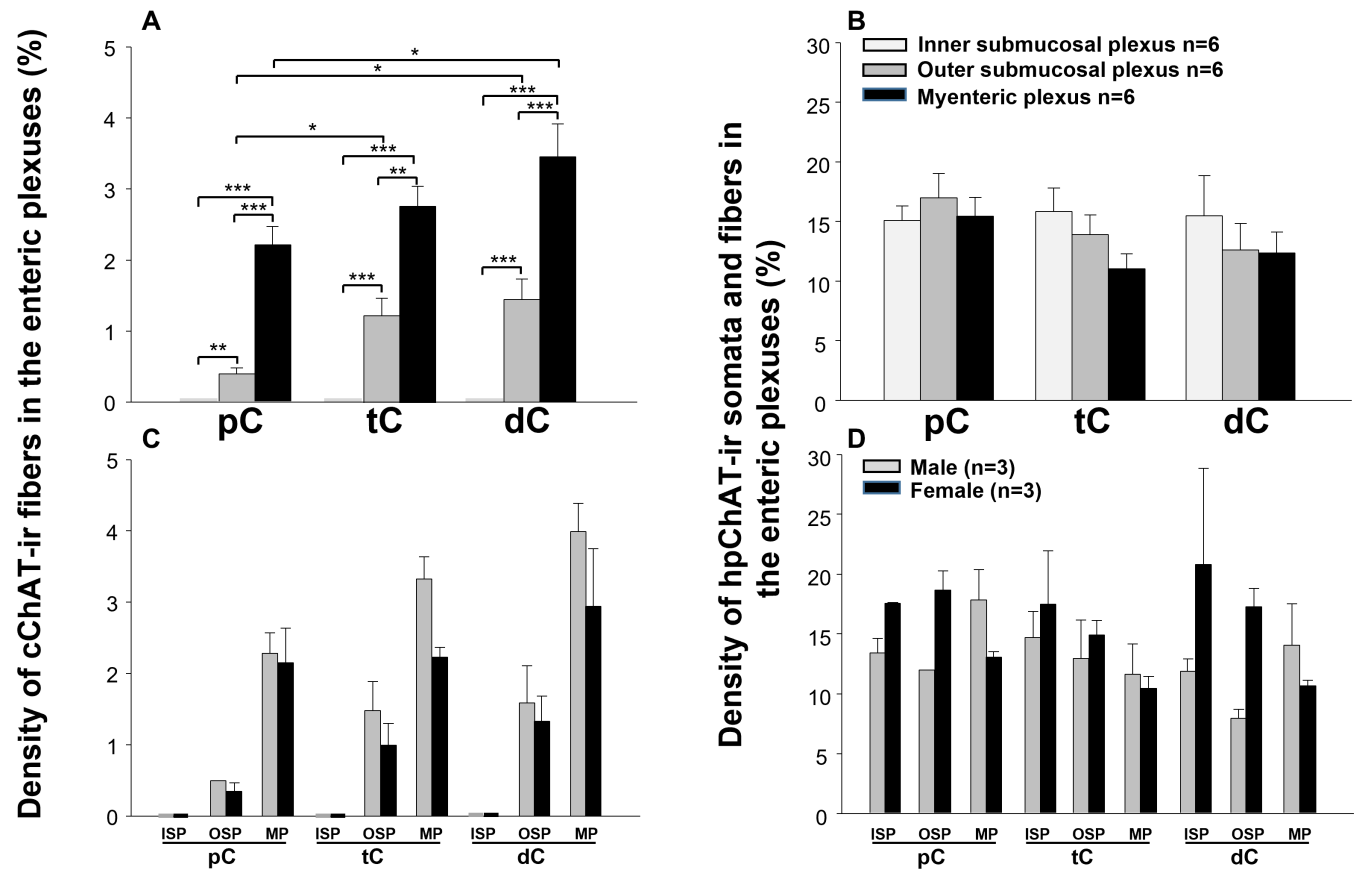
Quantitative analysis showed distinct densities of central and peripheral cholinergic innervation within the three enteric plexuses along different segments of the pig colon. The quantitation was carried out in 5–6 3D images with the volume X × Y × Z (axis) = 708 × 708 × 20–45 (μm^3^) acquired from the inner submucosal plexus, outer submucosal plexus and myenteric plexus of the proximal, transverse and distal colon (pC, dC, dC). The density of the cChAT immunoreactive (ir) fibers or hpChAT-ir fibers + somata was calculated and expressed as the percentages of their volumes in the delimited ganglion volume (v/v, %). In the upper panel **(A, B)**, each column represents the mean ± SEM of 6 pigs (3 of each male and female pooled together). In the lower panel **(C, D)**, each column represents mean ± SEM of 3 pigs (male and female separately). *, **, ***, *p* < 0.05, *p* < 0.01, <0.001.

The hpChAT immunostaining showed strong intensity and high sensitivity providing sharpest neuronal profile with fine fibers at a high resolution allowing us to quantitate the density of both cholinergic neuronal cell bodies and fibers separately from cChAT fibers as illustrated in Figure 6. hpChAT-ir fibers + somata and cChAT-ir fibers in the inner (Figure 6A), outer (Figure 6F) submucosal plexuses and myenteric plexus (Figure 6K) were digitally traced using ImarisSurface Function (Figures 6B,G,L) and extracted from these images separately (Figures 6C,H,M and Figures 6D,I,N). The ganglia containing cholinergic neurons/fibers were surfaced-marked (Figures 6E,J,O). The volume of traced nerve fibers, neurons and ganglia were automatically measured with Imaris 9.7. Quantitative analysis of the density indicates that the inner submucosal plexus had no cChAT-ir fibers in all three segments (Figure 7A). In the outer submucosal plexus, the density of cChAT-ir fibers in the transverse and distal colon was significantly 4.1- and 6.8-fold higher, respectively, than in the proximal colon. In the myenteric plexus, the pattern of cChAT-ir fiber density showed a linear increase trend from the proximal to distal colon (Figure 7A). No significant difference was found in the high density of hpChAT-ir fibers + somata among the plexuses and segments (Figure 7B). The patterns of cChAT or hpChAT-ir density in the three ganglia and segments were similar between males and females (Figures 7C,D).

### 3.5. Oral loperamide decreased the central and peripheral cholinergic innervation in the distal colonic myenteric plexus of pigs

**Figure 8:**
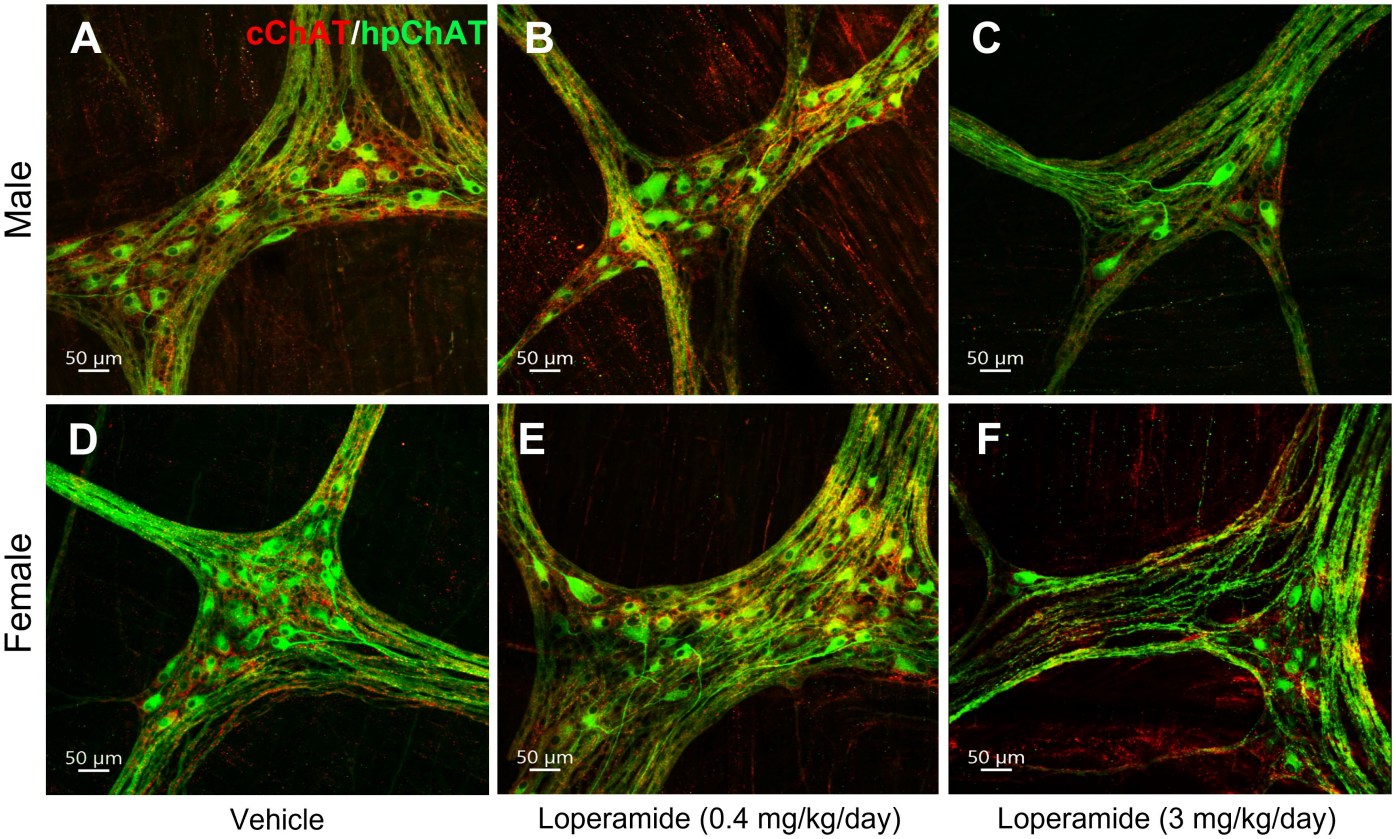
Double immunostaining of cChAT and hpChAT showed that oral loperamide (3 mg/kg/day, 4 weeks) decreased the central and peripheral cholinergic innervation in the distal colonic myenteric plexus of pigs. Double immunostaining with antibodies against a common type of choline acetyltransferase (cChAT, red) expressed preferentially in the central nervous system and a human peripheral form of choline acetyltransferase (hpChAT, green) was performed in the whole-mount preparations of the longitudinal muscle-myenteric plexus of the distal colon collected from pigs orally administrated for four weeks with vehicle control **(A,D)**, loperamide at 0.4 mg/kg/day **(B,E)** and 3 mg/kg/day **(C,F)**. **A–C**: male. **D–F**: female. Bar scale in each panel 50 μm.

**Figure 9:**
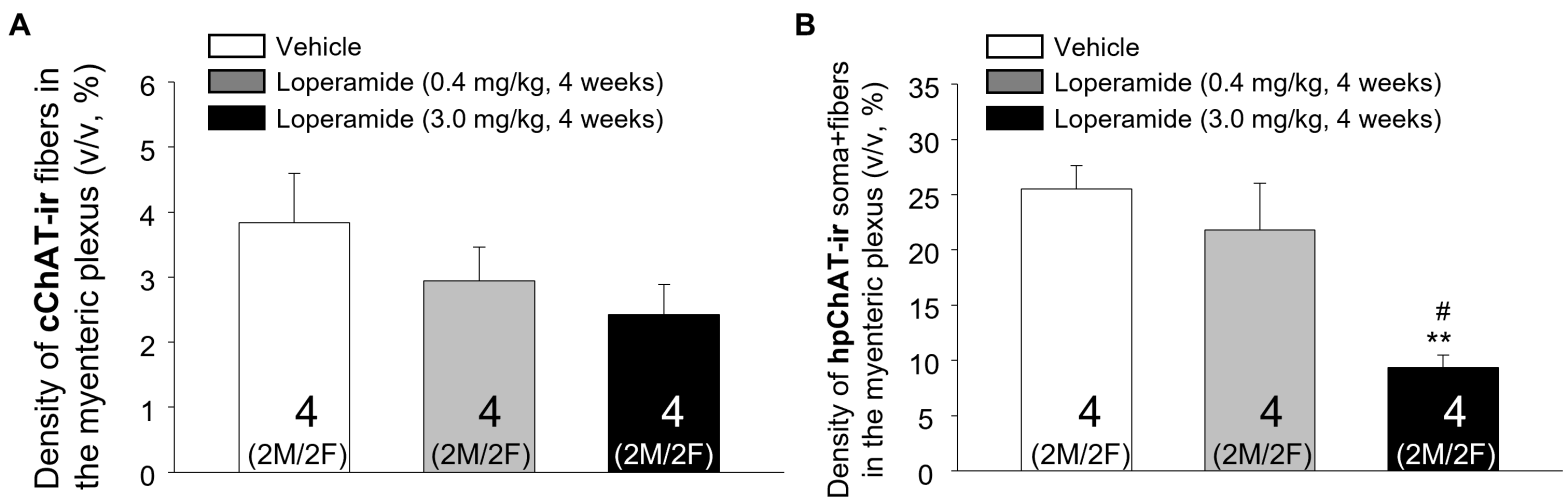
Computational quantitation with Imaris 9.7 showed that oral loperamide (3 mg/kg/day, 4 weeks) resulted in significant decrease of the peripheral cholinergic innervation in the distal colonic myenteric plexus of pigs. The central and peripheral cholinergic innervation were labeled with marker antibodies against common choline acetyltransferase (cChAT) and human peripheral form of ChAT (hpChAT) respectively in whole mount preparation of the distal colonic longitudinal muscle-myenteric plexus of pigs orally administrated for four weeks with vehicle controls and loperamide (0.4 mg and 3 mg/kg/day). Computational quantitation was performed with 6 of 3D images with the volume X × Y × Z (axis) = 708 × 708 × 20 (μm^3^)/image/animal. The cChAT immunoreactive (ir) fibers or hpChAT-ir fibers + somata were digitally traced with ImarisSurface functions. The densities of the cChAT-ir fibers **(A)** or hpChAT-ir fibers + somata **(B)** were calculated and expressed as the percentages of their volumes in the delimited ganglion volume (v/v, μm^3^/μm^3^, %). Each column represents mean ± SEM of 4 pigs (2 of each male and female pooled together). **p < 0.01 vs. control; ^#^*p* < 0.05 vs. low dose loperamide.

The daily oral administration of loperamide at 3 mg/kg for 4 weeks induced a decrease of cChAT-ir varicose fibers and hpChAT-ir fibers + somata in both males (Figures 8A–C) and females (Figures 8D–F) as displayed in the 3D images of whole mount preparation in the distal colonic myenteric plexus compared to vehicle. Quantitative analysis of myenteric plexus from males and females pooled together showed that loperamide (3 mg/kg) reduced the density of hpChAT-ir fibers + somata by 63% compared to vehicle group while loperamide at 0.4 mg/kg for 4 weeks had no effect (Figure 9B). The numbers of HuC/D labeled neurons in the myenteric plexus of the distal colon did not show significant differences among three groups (vehicle: 51337 ± 7,344, loperamide, 0.4 mg/kg/day: 44254 ± 3,485, 3 mg/kg/day: 38916 ± 467, mean ± SEM HuC/D-ir neurons/mm^3^ volume of ganglion, *n* = 4/group, *p* = 0.313).

cChAT-ir fibers in the pig distal colon showed a trend to have a dose-related decrease in their density induced by loperamide treatment which did not reach significance (*p* = 0.286) compared to the control group given vehicle (Figure 9A). The statical analysis of sex difference cannot be performed due to the small number of animals per sex (2 pigs/sex).

## 4. Discussion

The present study distinguished the central and peripheral cholinergic innervation of the colon and unraveled their spatial configuration in 3D images in the pig, a relevant translational model (Miller and Ullrey, 1987; Kararli, 1995; Patterson et al., 2008; Gonzalez et al., 2015). The quantitative analysis showed segmental differences among the inner and outer submucosal and myenteric plexuses in the central but not peripheral cholinergic innervation. The study also demonstrated that the chronic administration of loperamide, a preferential μ-opioid receptor agonist (DeHaven-Hudkins et al., 1999) decreased the peripheral myenteric cholinergic innervation of the distal colon. These findings were achieved by developing a set of novel approaches enabling to differentiate immunohistochemically the central cholinergic fibers from the peripheral cholinergic fibers plus somata in the enteric nervous system, to visualize their architecture and topography in 3D images of the intact muscularis externa generated with CLARITY tissue clearing technique and computationally quantifying the cholinergic densities in 3D images with Imaris 9.7 for Neuroscientists.

### 4.1. Validity of double immunolabeling of cChAT and hpChAT to distinguish central and peripheral cholinergic innervation in the pig colon enteric nervous system

The immunohistochemistry with antibody against choline acetyltransferase, an acetylcholine synthesizing enzyme, has been widely used to visualize cholinergic cell bodies and fibers in the central nervous system, however, it gave imprecise and faint staining of cholinergic cells and fibers in peripheral tissues (Kimura et al., 1980, 2007; Eckenstein et al., 1981; Levey et al., 1981; Anglade and Larabi-Godinot, 2010). A leap forward occurred with the identification of a ChAT mRNA splice variant in the rat peripheral nervous system lacking exons 6–9 in the coding region named rat peripheral form of ChAT (rpChAT) (Tooyama and Kimura, 2000; Kimura et al., 2007). rpChAT immunohistochemistry was useful to investigate the cholinergic enteric nervous system in rats (Nakajima et al., 2000; Yuan et al., 2005). Recently, we have successfully developed a novel mouse antiserum against the predicted human hpChAT using 14-amino acid sequence extending both wings before and after the junction of exons 5 and 10 of hpChAT protein (Bellier et al., 2019). The immunohistochemistry of hpChAT showed intense staining in many neuronal cells and fibers of human colon but not in human brain (Bellier et al., 2019). In the present study, we found that hpChAT-ir cells were fully co-labeled by the pan-neuronal marker HuC/D (Lin et al., 2002) in the pig colonic myenteric plexus. In addition, all neurons labeled by ChAT (AB144p), a ChAT antibody generated with antigen against human placental ChAT and commonly used to label enteric cholinergic neurons (Jiang et al., 2017; Li et al., 2019), were also co-labeled by hpChAT. Importantly, the hpChAT exhibited strong intensity and high sensitivity with a dilution up to 1:2,000–4,000 vs. 1:100–500 for the ChAT AB144p and provided sharp neuronal profile with the visualization of fine fibers at a high resolution in the pig myenteric ganglia. Further characterization of cChAT antibody, a central cholinergic marker in the rat brain (Kimura et al., 2007) and hpChAT antibodies using double labeling showed that cChAT labeled cholinergic neurons and fibers in the pig central nervous system structures without cross-reaction with hpChAT. This is demonstrated by the strong cChAT immunoreactivity in the fibers of the cervical vagal fascicles while the perineurium composed of fibrillar and microfibrillar collagens and fibronectin had no labeling. The cChAT antibody also labeled neuronal somata and fibers in the ventral horn of the pig sacral spinal cord while there was not hpChAT immunoreactivity visualized in these central nervous system structures. By contrast, in the whole mount preparation of pig colon myenteric plexus, there was dense hpChAT immunoreactivity in neurons and fibers, while cChAT immunohistochemistry was visualized only in the fibers with varicose morphology and dot-like structures but not in the somata. The few co-labeled fibers occasionally observed may represent peripheral cholinergic fibers considering the specificity of hpChAT to label exclusively the peripheral cholinergic system. However, it cannot be excluded that cChAT, a common type of ChAT in rats may have a weak cross-reaction with hpChAT in pig enteric nervous system. Collectively, these data ascertain the validity and selectivity of the double labeling with cChAT and hpChAT antibodies to distinguish central cholinergic axons and terminals from peripheral cholinergic neurons and fibers in the pig colon.

### 4.2. Spatial configuration of the two classes of cholinergic innervation and quantitative mapping of their distributions within the three layers of enteric plexuses along with the different segments of the pig colon

The central nerve fibers and peripheral neurons and fibers in the enteric nervous system form a 3D network which cannot be easily portrayed with 2D images acquired from tissue sections (Furness et al., 2014). However, the 3D imaging of the pig colon innervation faces challenges due to the thickness and opacity of colonic wall which restrict large volumes imaging with microscopic resolution (Tuchin et al., 1997; Tainaka et al., 2014). By developing a modified CLARITY protocol for the whole thickness of human colon combined with hpChAT immunohistochemistry, we previously demonstrated successfully the peripheral cholinergic innervation in 3D structure of the human sigmoid colon (Yuan et al., 2021a). In the present study, we further optimized the CLARITY protocol to speed up the tissue clearing by removing the mucosa, submucosa and serosa from the full thickness of the pig proximal colon and trimmed the muscularis externa into a small volume (1 × 1 × 0.2 cm) after fixation with 4% paraformaldehyde alone. These steps cut down by over a half the clearing time required in our original protocol (Yuan et al., 2021a). The smaller volume also resulted in less light scattering and clearer imaging allowing us to show myenteric cholinergic neurons (hpChAT labeled) in ganglia closely surrounded by central cholinergic (cChAT-ir) varicose fibers with dot like structures, nerve terminals in the pig proximal colon. These findings provide neuroanatomical evidence that central efferent cholinergic fibers target myenteric neurons in the pig colon. This is consistent with the report showing that in the guinea pig distal colon, most of the effects of parasympathetic input are mediated via enteric neuronal circuits, particularly myenteric neurons which are the major recipient of central fibers involved in gut motility rather than direct input to effector cells (Chen et al., 2015).

The development of an approach to computationally quantitate the density of cChAT-ir fibers and hpChAT-ir fibers and somata in 3D images of the whole mount enteric preparation had the advantage to distinguish two classes of cholinergic fibers and hpChAT-ir whole cell bodies and processes without the loss of fibers as occurring in 2D. With regards to the inner submucosal plexus, the quantitative analysis indicated that there is no cChAT-ir fibers in the proximal, transverse and distal colon while there was a high density of hpChAT-ir somata and fibers similar in these three segments. The inner submucosal plexus was localized in the submucosa close to the lamina muscularis mucosae, and not in the mucosa, therefore it is unlikely that the removal of mucosa damaged the visualization of cChAT fibers. In addition, under these conditions, we found extrinsic sympathetic nerve fibers labeled by tyrosine hydroxylase in the pig inner submucosal plexus (Yuan et al., unpublished observations). These findings provide evidence that the cholinergic innervation in the inner submucosal plexus of the pig colon arise from the peripheral cholinergic system with no direct input from the central cholinergic fibers. By contrast, the outer submucosal plexus is innervated by cChAT fibers pointing to this submucosal plexus as the recipient of central cholinergic input. The mucosa is primarily controlled by submucosal ganglia with cholinergic neurons being secretomotor (Furness, 2008). We previously reported that central parasympathetic stimulation of the colon by corticotropin releasing factor injected into the 4th ventricle induced diarrhea in rats which was correlated with a greater number of activated submucosal neurons in the distal compared in the proximal colon (Yuan et al., 2021b). These functional data may have a bearing with the 3.7-fold higher (*p* < 0.05) density of cChAT found in the outer submucosal plexus of the distal than proximal colon. In the myenteric plexus, the density of cChAT was significantly higher than in the outer submucosal plexus in each segment and showed a linear increase from the proximal to distal colon being 1.6-fold higher in the distal than the proximal colon (*p* < 0.05). Central cholinergic fibers in the myenteric plexus are consistent with the importance of central parasympathetic innervation in the control of expulsion while in the proximal colon is specialized for the retention and mixing of luminal contents (Petto et al., 2015). By contrast, the peripheral cholinergic innervation (hpChAT positive fibers + somata) was dense and had no significant differences among the plexuses and segments.

It is to note that the results related to the density of central and peripheral cholinergic innervation in the pig colonic segments have some limitations. First, we could not differentiate whether hpChAT-ir fibers in the distal colon are only of intrinsic enteric origins. While cholinergic nerve cell bodies in sacral segments of the spinal cord project directly to the distal colon, many of these axons also synapse onto nerve cell bodies located within the extrinsic inferior hypogastric ganglia which in turn project to the distal colon (Fukai and Fukuda, 1985; Keast and de Groat, 1989; Olsson et al., 2006). Moreover, within the inferior hypogastric ganglia, preganglionic sympathetic fibers are thought to synapse onto postganglionic cholinergic fibers (Luckensmeyer and Keast, 1995; Olsson et al., 2006), allowing pathways for sympathetic-cholinergic input to the colon. Previous studies indicated that rpChAT-immunoreactivity can be detected in peripheral cholinergic postganglionic neurons (Bellier and Kimura, 2011; Koga et al., 2013). Whether hpChAT immunoreactivity can be identified in neurons of the pig inferior hypogastric ganglia projecting to the distal colon needs to be assessed to ascertain that the hpChAT fibers are solely of intramural origin. Second, the enteric hpChAT cholinergic innervation in the proximal and transverse colon reflects only the area between the two taeniae where the colon wall is covered with a relatively thin layer of longitudinal muscle and easier to perform the CLARITY and whole mount preparation. In human colon whole mount preparation, a report indicated that the myenteric plexus is 50% denser and 30% wider in areas underneath the tenia than those between the tenia coli, however, the density and size of the dihydronicotinamide adenine dinucleotide phosphate diaphorase (NADPH-d) positive neurons were similar between underneath and between the taeniae (Hanani et al., 2012). Whether similar observations applied to the pig colonic myenteric plexus underneath the tenia remained to be established. Likewise, the cChAT cholinergic innervation might be denser at the point of entrance of nerves presumably near the tenia mesocolica, however this information is lacking in the pig colon and could not be assessed. Lastly the absence of sex difference in the central and peripheral cholinergic innervation should be put in the context that male pigs have been castrated early on at postnatal day 7.

The ganglia labeled with hpChAT antibody showed marked differences in the morphology and density among the three plexuses with small but of high density in the inner submucosal plexus, sparse with a wider meshwork in the outer submucosal plexus and heterogeneous and dense in the myenteric plexus. These findings agree with a report in pig colonic enteric ganglia labeled with HuC/D antibody (Petto et al., 2015). This reflects different types of cholinergic neurons located in the myenteric ganglia including excitatory motor neurons, ascending and descending interneurons, intrinsic primary afferent, intestinal fugal neurons and secretomotor neurons (Furness, 2000; Humenick et al., 2021; Sharkey and Mawe, 2023).

### 4.3. Loperamide reduced the cholinergic innervation of the pig colon myenteric plexus

Repeated peripheral administration of loperamide at the doses of 4–5 mg/kg body weight is widely used to induce constipation in healthy rodents (Kim et al., 2019a,b; Ma et al., 2021; Wang et al., 2023). Recently, we developed a loperamide model of constipation in healthy pigs using the oral dose of 3 mg/kg/day for four weeks. This regimen results in altered colonic motility patterns and decreased gastrointestinal transit and fecal water content with no direct signs of intolerance or toxicity (Larauche et al., 2022). In the present study, a similar administration of loperamide reduced by 63% the density of peripheral cholinergic innervation (hpChAT-ir neurons and fibers) in the pig distal colon myenteric plexus. By contrast, there was no significant changes in the extrinsic cholinergic density (cChAT-ir fibers). These findings are in keeping with the established peripheral action of loperamide which does not cross the blood brain barrier (DeHaven-Hudkins et al., 1999; Baker, 2007). The reduction of the density of hpChAT-ir neurons and fibers is likely to due to the decrease in peripheral cholinergic innervation as the number of HuC/D labeled myenteric neurons was not significantly changed. Such findings provide anatomical support underpinning the alterations of cholinergic transmission previously reported after loperamide treatment-induced constipation (Kim et al., 2019a,b; Wang et al., 2023). In naïve rats, loperamide reduced acetylcholine esterase activity and the expression of muscarinic cholinergic M2 and M3 receptors in the colon (Kim et al., 2019a,b) and in mice, the level of acetylcholine in the colonic tissue was decreased after repeated loperamide-induced functional constipation (Wang et al., 2023). Likewise, in healthy isolated tenia coli muscle, loperamide decreased the release of acetylcholine produced by electrical field stimulation (Burleigh, 1988). A recent report using human inter-tenia circular muscle strips also indicates that loperamide had no direct effect on neuromuscular function supporting an action primarily on enteric neurons (Heitmann et al., 2022). In the clinical setting, loperamide is used for the treatment of acute diarrhea at the therapeutic doses of 2–4 mg/subject (Vearrier and Grundmann, 2021). Such a dosage bears similarity with the 0.4 mg/kg/day of loperamide tested in our study that did not change the myenteric cholinergic innervation of the distal colon in healthy pigs. However, under conditions of abuse, intake frequently reaches more than 200 mg/day (Vearrier and Grundmann, 2021) resulting in constipation and bowel dysfunction (Dorn et al., 2014; Laroche et al., 2017) becoming a growing concern in the management of opioid side effects (Farmer et al., 2019).

In conclusion, this study validates the use of two specific cChAT and hpChAT antibodies to map the cholinergic innervation of the inner and outer submucosal and myenteric plexuses in the three segments of pig colon and to simultaneously differentiate the central (cChAT) from peripheral (hpChAT) cholinergic innervation. The 3D images generated from CLARITY-cleared pig colonic tissue samples demonstrate that some cChAT-ir varicose fibers are surrounding hpChAT-ir myenteric neurons with dot like structures supporting central and myenteric cholinergic interactions while the inner submucosal plexus did not receive direct central cholinergic input throughout the pig colon. Quantitative analysis reveals that chronic oral treatment with the peripheral mu opioid agonist, loperamide, results in a significant decrease in the peripheral but not central myenteric cholinergic innervation of the distal colon which may play a role in the development of opioid-induced constipation. These data provide a morphological basis for the evaluation of cholinergic alterations of the enteric nervous system associated with functional colonic disorders (Holland et al., 2021).

## Data availability statement

The authors confirm that the data supporting the findings of this study are available within the article. A dataset of characterizing of 30 antibodies (including those used in this study) in immunostaining with whole mount preparations of enteric plexus, CLARITY cleared intact samples and/or cryostat sections of pig colon was published in the Pennsieve.io (DOI: 10.26275/of13-iokw). A protocol describing a step-by-step computational workflow for the density measurement of cholinergic innervation in three-dimensional (3D) images of the pig colonic enteric nervous system (ENS) with Imaris 9.7 for neuroscientists was published in the protocols.io (dx.doi.org/10.17504/protocols.io.q26g7pry9gwz/v1).

## Ethics statement

The animal studies were approved by University of California at Los Angeles (UCLA) Institutional Animal Care and Use Committee, Chancellor’s Animal Research Committee (ARC) (approval protocol 2018-074-01). The studies were conducted in accordance with the local legislation and institutional requirements. Written informed consent was not obtained from the owners for the participation of their animals in this study because the pigs were purchased from S&S Farms (Farms, Ramona, CA).

## Author contributions

P-QY and YT: design the study and draft the manuscript. P-QY, TL, MM, ML, and KA performed the experiments. P-QY and TL: data acquisition and interpretation. MM and J-PB: give input on the written manuscript. All authors listed provide final approval for publication.

## Funding

This research was supported by the NIH SPARC OT2 grant OD024899 (P-QY, YT, and MM), NIHDDK Grant P30-DK-41301 (YT), U01NS113871 (MM, P-QY, ML, and KA), Veteran Administration Research Career Scientist Award (YT).

## Conflict of interest

J-PB has a registered Japanese patent (7176724). Antibody for detecting human peripheral cholinergic nerve (2022).

The remaining authors declare that the research was conducted in the absence of any commercial or financial relationships that could be construed as a potential conflict of interest.

## Publisher’s note

All claims expressed in this article are solely those of the authors and do not necessarily represent those of their affiliated organizations, or those of the publisher, the editors and the reviewers. Any product that may be evaluated in this article, or claim that may be made by its manufacturer, is not guaranteed or endorsed by the publisher.

## Footnotes

^1^https://imaris.oxinst.com/products/imaris-for-neuroscientists

^2^dx.doi.org/10.17504/protocols.io.q26g7pry9gwz/v1

## References

[ref700] Anglade P. Larabi-Godinot Y. (2010). Historical landmarks in the histochemistry of the cholinergic synapse: Perspectives for future researches. Biomed Res 31, 1–12. doi: 10.2220/biomedres.31.1, PMID: 20203414

[ref1] Baker D. E. (2007). Loperamide: a pharmacological review. Rev. Gastroenterol. Disord. 7, S11–S18. 18192961

[ref2] Bassotti G. Chiarioni G. Imbimbo B. P. Betti C. Bonfante F. Vantini I.. (1993). Impaired colonic motor response to cholinergic stimulation in patients with severe chronic idiopathic (slow transit type) constipation. Dig. Dis. Sci. 38, 1040–1045. doi: 10.1007/BF01295719, PMID: 8508698

[ref3] Bellier J. P. Kimura H. (2011). Peripheral type of choline acetyltransferase: biological and evolutionary implications for novel mechanisms in cholinergic system. J. Chem. Neuroanat. 42, 225–235. doi: 10.1016/j.jchemneu.2011.02.005 21382474

[ref4] Bellier J. P. Yuan P. Q. Mukaisho K. Tooyama I. Taché Y. Kimura H. (2019). A novel antiserum against a predicted human peripheral choline acetyltransferase (hpchat) for labeling structures in human colon. Front. Neuroanat. 13:37. doi: 10.3389/fnana.2019.00037, PMID: 31040770 PMC6476985

[ref5] Berthoud H. R. Carlson N. R. Powley T. L. (1991). Topography of efferent vagal innervation of the rat gastrointestinal tract. Am. J. Phys. 260, R200–R207. doi: 10.1152/ajpregu.1991.260.1.R200, PMID: 1992820

[ref6] Bharucha A. E. Pemberton J. H. Locke G. R. 3rd. (2013). American Gastroenterological Association technical review on constipation. Gastroenterology 144, 218–238. doi: 10.1053/j.gastro.2012.10.028, PMID: 23261065 PMC3531555

[ref7] Brehmer A. Rupprecht H. Neuhuber W. (2010). Two submucosal nerve plexuses in human intestines. Histochem. Cell Biol. 133, 149–161. doi: 10.1007/s00418-009-0657-2, PMID: 19911189

[ref8] Brown D. R. Timmermans J. P. (2004). Lessons from the porcine enteric nervous system. Neurogastroenterol. Motil. 16, 50–54. doi: 10.1111/j.1743-3150.2004.00475.x, PMID: 15066005

[ref9] Browning K. N. Travagli R. A. (2014). Central nervous system control of gastrointestinal motility and secretion and modulation of gastrointestinal functions. Compr. Physiol. 4, 1339–1368. doi: 10.1002/cphy.c130055 25428846 PMC4858318

[ref10] Burleigh D. E. (1988). Opioid and non-opioid actions of loperamide on cholinergic nerve function in human isolated colon. Eur. J. Pharmacol. 152, 39–46. doi: 10.1016/0014-2999(88)90833-3, PMID: 2850201

[ref11] Camilleri M. Drossman D. A. Becker G. Webster L. R. Davies A. N. Mawe G. M. (2014). Emerging treatments in neurogastroenterology: a multidisciplinary working group consensus statement on opioid-induced constipation. Neurogastroenterol. Motil. 26, 1386–1395. doi: 10.1111/nmo.12417, PMID: 25164154 PMC4358801

[ref12] Chen B. N. Sharrad D. F. Hibberd T. J. Zagorodnyuk V. P. Costa M. Brookes S. J. (2015). Neurochemical characterization of extrinsic nerves in myenteric ganglia of the guinea pig distal colon. J. Comp. Neurol. 523, 742–756. doi: 10.1002/cne.23704, PMID: 25380190

[ref13] Chung K. Deisseroth K. (2013). CLARITY for mapping the nervous system. Nat. Methods 10, 508–513. doi: 10.1038/nmeth.2481 23722210

[ref14] Dart R. C. Severtson S. G. Bucher-Bartelson B. (2015). Trends in opioid analgesic abuse and mortality in the United States. N. Engl. J. Med. 372, 241–248. doi: 10.1056/NEJMsa1406143 25587948

[ref15] DeHaven-Hudkins D. L. Burgos L. C. Cassel J. A. Daubert J. D. DeHaven R. N. Mansson E.. (1999). Loperamide (ADL 2-1294), an opioid antihyperalgesic agent with peripheral selectivity. J. Pharmacol. Exp. Ther. 289, 494–502. PMID: 10087042

[ref16] Dorn S. Lembo A. Cremonini F. (2014). Opioid-induced bowel dysfunction: epidemiology, pathophysiology, diagnosis, and initial therapeutic approach. Am. J. Gastroenterol. Suppl. 2, 31–37. doi: 10.1038/ajgsup.2014.7 25207610

[ref17] Eckenstein F. Barde Y. A. Thoenen H. (1981). Production of specific antibodies to choline acetyltransferase purified from pig brain. Neuroscience 6, 993–1000. doi: 10.1016/0306-4522(81)90065-8, PMID: 7279221

[ref18] Farmer A. D. Drewes A. M. Chiarioni G. De Giorgio R. O'Brien T. Morlion B.. (2019). Pathophysiology and management of opioid-induced constipation: European expert consensus statement. United European Gastroenterol. J. 7, 7–20. doi: 10.1177/2050640618818305 PMC6374852 30788113

[ref19] Fukai K. Fukuda H. (1985). Three serial neurons in the innervation of the colon by the sacral parasympathetic nerve of the dog. J. Physiol. 362, 69–78. doi: 10.1113/jphysiol.1985.sp015663, PMID: 4020695 PMC1192882

[ref20] Furness J. B. (2000). Types of neurons in the enteric nervous system. J. Auton. Nerv. Syst. 81, 87–96. doi: 10.1016/S0165-1838(00)00127-2 10869706

[ref21] Furness J. B. (2008). The enteric nervous system: normal functions and enteric neuropathies. Neurogastroenterol. Motil. 20, 32–38. doi: 10.1111/j.1365-2982.2008.01094.x 18402640

[ref22] Furness J. B. Callaghan B. P. Rivera L. R. Cho H. (2014). The enteric nervous system and gastrointestinal innervation: integrated local and central control. Adv. Exp. Med. Biol. 817, 39–71. doi: 10.1007/978-1-4939-0897-4_3, PMID: 24997029

[ref23] Gagnon B. Scott S. Nadeau L. Lawlor P. G. (2015). Patterns of community-based opioid prescriptions in people dying of cancer. J. Pain Symptom Manag. 49, 36–44.e1. doi: 10.1016/j.jpainsymman.2014.05.015 24945491

[ref24] Galligan J. J. (2002). Pharmacology of synaptic transmission in the enteric nervous system. Curr. Opin. Pharmacol. 2, 623–629. doi: 10.1016/S1471-4892(02)00212-6 12482723

[ref25] Gonella J. Bouvier M. Blanquet F. (1987). Extrinsic nervous control of motility of small and large intestines and related sphincters. Physiol. Rev. 67, 902–961. doi: 10.1152/physrev.1987.67.3.902, PMID: 3299412

[ref26] Gonzalez L. Moeser A. J. Blikslager A. T. (2015). Porcine models of digestive disease: the future of large animal translational research. Transl. Res. 166, 12–27. doi: 10.1016/j.trsl.2015.01.004, PMID: 25655839 PMC4458388

[ref27] Gregorian T. Lewis J. Tsu L. (2017). Opioid-induced constipation: clinical guidance and approved therapies. Pharm 42, 15–19.

[ref28] Hanani M. Grossman S. Nissan A. Eid A. (2012). Morphological and quantitative study of the myenteric plexus in the human tenia coli. Anat. Rec. (Hoboken) 295, 1321–1326. doi: 10.1002/ar.22511, PMID: 22678779

[ref29] Hao M. M. Bornstein J. C. Young H. M. (2013). Development of myenteric cholinergic neurons in ChAT-Cre;R26R-YFP mice. J. Comp. Neurol. 521, 3358–3370. doi: 10.1002/cne.23354 23649862

[ref30] Hedemann M. S. Kristiansen E. Brunsgaard G. (2002). Morphology of the large intestine of the pig: haustra versus taenia. Ann. Anat. 184, 401–403. doi: 10.1016/S0940-9602(02)80065-6, PMID: 12201052

[ref31] Heitmann P. T. Keightley L. Wiklendt L. Wattchow D. A. Brookes S. S. J. Spencer N. J.. (2022). The effects of loperamide on excitatory and inhibitory neuromuscular function in the human colon. Neurogastroenterol. Motil. 34:e14442. doi: 10.1111/nmo.14442, PMID: 36054796

[ref32] Higginson I. J. Gao W. (2012). Opioid prescribing for cancer pain during the last 3 months of life: associated factors and 9-year trends in a nationwide United Kingdom cohort study. J. Clin. Oncol. 30, 4373–4379. doi: 10.1200/JCO.2012.42.0919, PMID: 23109701

[ref33] Ho A. Lievore A. Patierno S. Kohlmeier S. E. Tonini M. Sternini C. (2003). Neurochemically distinct classes of myenteric neurons express the mu-opioid receptor in the guinea pig ileum. J. Comp. Neurol. 458, 404–411. doi: 10.1002/cne.10606, PMID: 12619074

[ref34] Holland A. M. Bon-Frauches A. C. Keszthelyi D. Melotte V. Boesmans W. (2021). The enteric nervous system in gastrointestinal disease etiology. Cell. Mol. Life Sci. 78, 4713–4733. doi: 10.1007/s00018-021-03812-y, PMID: 33770200 PMC8195951

[ref35] Hoyle C. H. Burnstock G. (1989). Neuronal populations in the submucous plexus of the human colon. J. Anat. 166, 7–22. PMID: 2621148 PMC1256735

[ref36] Humenick A. Chen B. N. Wattchow D. A. Zagorodnyuk V. P. Dinning P. G. Spencer N. J.. (2021). Characterization of putative interneurons in the myenteric plexus of human colon. Neurogastroenterol. Motil. 33:e13964. doi: 10.1111/nmo.13964, PMID: 32839997 PMC7772282

[ref37] Hunter D. A. Pan D. Wood M. D. Snyder-Warwick A. K. Moore A. M. Feldman E. Z. L.. (2020). Design-based stereology and binary image histomorphometry in nerve assessment. J. Neurosci. Methods 336:108635. doi: 10.1016/j.jneumeth.2020.108635, PMID: 32070676 PMC8045463

[ref38] Jiang Y. Dong H. Eckmann L. Hanson E. M. Ihn K. C. Mittal R. K. (2017). Visualizing the enteric nervous system using genetically engineered double reporter mice: comparison with immunofluorescence. PLoS One 12:e0171239. doi: 10.1371/journal.pone.0171239, PMID: 28158225 PMC5291392

[ref39] Kararli T. T. (1995). Comparison of the gastrointestinal anatomy, physiology, and biochemistry of humans and commonly used laboratory animals. Biopharm. Drug Dispos. 16, 351–380. doi: 10.1002/bdd.2510160502, PMID: 8527686

[ref40] Keast J. R. de Groat W. C. (1989). Immunohistochemical characterization of pelvic neurons which project to the bladder, colon, or penis in rats. J. Comp. Neurol. 288, 387–400. doi: 10.1002/cne.902880303 2571623

[ref41] Kim J. E. Park J. W. Kang M. J. Choi H. J. Bae S. J. Choi Y. S.. (2019b). Anti-inflammatory response and muscarinic cholinergic regulation during the laxative effect of *Asparagus Cochinchinensis* in loperamide-induced constipation of SD rats. Int. J. Mol. Sci. 20:946. doi: 10.3390/ijms20040946, PMID: 30795644 PMC6412595

[ref42] Kim J. E. Park J. W. Kang M. J. Choi H. J. Bae S. J. Choi Y.. (2019a). Laxative effect of spicatoside a by cholinergic regulation of enteric nerve in loperamide-induced constipation: ICR mice model. Molecules 24:896. doi: 10.3390/molecules24050896, PMID: 30836659 PMC6429089

[ref43] Kimura S. Bellier J. P. Matsuo A. Tooyama I. Kimura H. (2007). The production of antibodies that distinguish rat choline acetyltransferase from its splice variant product of a peripheral type. Neurochem. Int. 50, 251–255. doi: 10.1016/j.neuint.2006.08.011 17011076

[ref44] Kimura H. McGeer P. L. Peng F. McGeer E. G. (1980). Choline acetyltransferase-containing neurons in rodent brain demonstrated by immunohistochemistry. Science 208, 1057–1059. doi: 10.1126/science.6990490, PMID: 6990490

[ref45] Koga T. Bellier J. P. Kimura H. Tooyama I. (2013). Immunoreactivity for choline acetyltransferase of peripheral-type (pChAT) in the trigeminal ganglion neurons of the non-human primate *Macaca fascicularis*. Acta Histochem. Cytochem. 46, 59–64. doi: 10.1267/ahc.12044, PMID: 23720604 PMC3661780

[ref46] Lai H. M. Liu A. K. L. Ng W.-L. DeFelice J. Lee W. S. Li H.. (2016). Rationalization and validation of an acrylamide-free procedure in three-dimensional histological imaging. PLoS One 11:e0158628. doi: 10.1371/journal.pone.0158628, PMID: 27359336 PMC4928791

[ref47] Larauche M. H. Atmani K. Navalgund A. R. Wang Y. Axelrod L. Liu W.. (2022). Development and characterization of an opioid-induced constipation (OIC) model in swine: use of G-tech wireless non-invasive patches to monitor colonic myoelectrocal activity (CMA). Gastroenterology 162, S-703–S-704. doi: 10.1016/S0016-5085(22)61649-4

[ref48] Laroche F. Meissner W. Morlion B. (2017). Opioid-induced constipation and bowel dysfunction: a clinical guideline. Pain Med. 18, 1837–1863. doi: 10.1093/pm/pnw255 28034973 PMC5914368

[ref49] Levey A. I. Aoki M. Fitch F. W. Wainer B. H. (1981). The production of monoclonal antibodies reactive with bovine choline acetyltransferase. Brain Res. 218, 383–387. doi: 10.1016/0006-8993(81)91316-0 7272744

[ref50] Li Z. Hao M. M. Van den Haute C. Baekelandt V. Boesmans W. Vanden Berghe P. (2019). Regional complexity in enteric neuron wiring reflects diversity of motility patterns in the mouse large intestine. elife 8:e42914. doi: 10.7554/eLife.42914, PMID: 30747710 PMC6391068

[ref51] Li T. Morselli M. Su T. Million M. Larauche M. Pellegrini M.. (2023). Comparative transcriptomics reveals highly conserved regional programs between porcine and human colonic enteric nervous system. Commun. Biol. 6:98. doi: 10.1038/s42003-023-04478-x, PMID: 36693960 PMC9872754

[ref52] Lin Z. Gao N. Hu H. Z. Liu S. Gao C. Kim G.. (2002). Immunoreactivity of Hu proteins facilitates identification of myenteric neurones in guinea-pig small intestine. Neurogastroenterol. Motil. 14, 197–204. doi: 10.1046/j.1365-2982.2002.00317.x, PMID: 11975720

[ref53] Luckensmeyer G. B. Keast J. R. (1995). Immunohistochemical characterisation of sympathetic and parasympathetic pelvic neurons projecting to the distal colon in the male rat. Cell Tissue Res. 281, 551–559. doi: 10.1007/BF00417873 7553774

[ref54] Lunney J. K. Van Goor A. Walker K. E. Hailstock T. Franklin J. Dai C. (2021). Importance of the pig as a human biomedical model. Sci. Transl. Med. 13:eabd5758. doi: 10.1126/scitranslmed.abd5758 34818055

[ref55] Ma L. Qu Z. Xu L. Han L. Han Z. G. He J.. (2021). 7,8-Dihydroxyflavone enhanced colonic cholinergic contraction and relieved loperamide-induced constipation in rats. Dig. Dis. Sci. 66, 4251–4262. doi: 10.1007/s10620-020-06817-y, PMID: 33528684

[ref56] Meerschaert K. A. Davis B. M. Smith-Edwards K. M. (2022). New insights on extrinsic innervation of the enteric nervous system and non-neuronal cell types that influence colon function. Adv. Exp. Med. Biol. 1383, 133–139. doi: 10.1007/978-3-031-05843-1_13, PMID: 36587153

[ref57] Miller E. R. Ullrey D. E. (1987). The pig as a model for human nutrition. Annu. Rev. Nutr. 7, 361–382. doi: 10.1146/annurev.nu.07.070187.002045 3300739

[ref58] Mori Y. Shibasaki Y. Matsumoto K. Shibasaki M. Hasegawa M. Wang E.. (2013). Mechanisms that underlie μ-opioid receptor agonist-induced constipation: differential involvement of μ-opioid receptor sites and responsible regions. J. Pharmacol. Exp. Ther. 347, 91–99. doi: 10.1124/jpet.113.204313, PMID: 23902939

[ref59] Müller-Lissner S. Bassotti G. Coffin B. Drewes A. M. Breivik H. Eisenberg E.. (2017). Opioid-induced constipation and bowel dysfunction: A clinical guideline. Pain Med. 18, 1837–1863. doi: 10.1093/pm/pnw255, PMID: 28034973 PMC5914368

[ref60] Murphy W. J. Larkin D. M. Everts-van der Wind A. Bourque G. Tesler G. Auvil L.. (2005). Dynamics of mammalian chromosome evolution inferred from multispecies comparative maps. Science 309, 613–617. doi: 10.1126/science.1111387, PMID: 16040707

[ref61] Nakajima K. Tooyama I. Yasuhara O. Aimi Y. Kimura H. (2000). Immunohistochemical demonstration of choline acetyltransferase of a peripheral type (pChAT) in the enteric nervous system of rats. J. Chem. Neuroanat. 8, 31–40. doi: 10.1016/s0891-0618 10708917

[ref62] Olsson C. Chen B. N. Jones S. Chataway T. K. Costa M. Brookes S. J. (2006). Comparison of extrinsic efferent innervation of guinea pig distal colon and rectum. J. Comp. Neurol. 496, 787–801. doi: 10.1002/cne.20965, PMID: 16628614

[ref63] Pappagallo M. (2001). Incidence, prevalence, and management of opioid bowel dysfunction. Am. J. Surg. 182, S11–S18. doi: 10.1016/S0002-9610(01)00782-6 11755892

[ref64] Patterson J. K. Lei X. G. Miller D. D. (2008). The pig as an experimental model for elucidating the mechanisms governing dietary influence on mineral absorption. Exp. Biol. Med. 233, 651–664. doi: 10.3181/0709-MR-262 18408137

[ref65] Petto C. Gäbel G. Pfannkuche H. (2015). Architecture and chemical coding of the inner and outer submucous plexus in the colon of piglets. PLoS One 10:e0133350. doi: 10.1371/journal.pone.0133350, PMID: 26230272 PMC4521800

[ref66] Schneider D. A. Galligan J. J. (2000). Presynaptic nicotinic acetylcholine receptors in the myenteric plexus of guinea pig intestine. Am. J. Physiol. Gastrointest. Liver Physiol. 279, G528–G535. doi: 10.1152/ajpgi.2000.279.3.G528, PMID: 10960351

[ref67] Schneider S. Wright C. M. Heuckeroth R. O. (2019). Unexpected roles for the second brain: enteric nervous system as master regulator of bowel function. Annu. Rev. Physiol. 81, 235–259. doi: 10.1146/annurev-physiol-021317-121515 30379617

[ref68] Sharkey K. A. Mawe G. M. (2023). The enteric nervous system. Physiol. Rev. 103, 1487–1564. doi: 10.1152/physrev.00018.2022, PMID: 36521049 PMC9970663

[ref69] Spencer N. J. Dinning P. G. Brookes S. J. Costa M. (2016). Insights into the mechanisms underlying colonic motor patterns. J. Physiol. 594, 4099–4116. doi: 10.1113/JP271919, PMID: 26990133 PMC4967752

[ref70] Stevens C. E. Argenzio R. A. Roberts M. C. (1986). Comparative physiology of the mammalian colon and suggestions for animal models of human disorders. Clin. Gastroenterol. 15, 763–785. PMID: 3536208

[ref71] Tainaka K. Kubota S. I. Suyama T. Q. Susaki E. A. Perrin D. Ukai-Tadenuma M.. (2014). Whole-body imaging with single-cell resolution by tissue decolorization. Cells 159, 911–924. doi: 10.1016/j.cell.2014.10.034, PMID: 25417165

[ref72] Tao J. Campbell J. N. Tsai L. T. Wu C. Liberles S. D. Lowell B. B. (2021). Highly selective brain-to-gut communication via genetically defined vagus neurons. Neuron 109, 2106–2115.e4. doi: 10.1016/j.neuron.2021.05.004, PMID: 34077742 PMC8273126

[ref73] Thomas J. W. Touchman J. W. Blakesley R. W. Bouffard G. G. Beckstrom-Sternberg S. M. Margulies E. H.. (2003). Comparative analyses of multi-species sequences from targeted genomic regions. Nature 424, 788–793. doi: 10.1038/nature01858, PMID: 12917688

[ref74] Timmermans J. P. Adriaensen D. Cornelissen W. Scheuermann D. W. (1997). Structural organization and neuropeptide distribution in the mammalian enteric nervous system, with special attention to those components involved in mucosal reflexes. Comp. Biochem. Physiol. Part A. Physiol. 118, 331–340. doi: 10.1016/s0300-9629(96)00314-3, PMID: 9366065

[ref75] Timmermans J. P. Hens J. Adriaensen D. (2001). Outer submucous plexus: an intrinsic nerve network involved in both secretory and motility processes in the intestine of large mammals and humans. Anat. Rec. 262, 71–78. doi: 10.1002/1097-0185(20010101)262:1<71::AID-AR1012>3.0.CO;2-A, PMID: 11146430

[ref76] Tooyama I. Kimura H. (2000). A protein encoded by an alternative splice variant of choline acetyltransferase mRNA is localized preferentially in peripheral nerve cells and fibers. J. Chem. Neuroanat. 17, 217–226. doi: 10.1016/S0891-0618(99)00043-5, PMID: 10697248

[ref77] Tuchin V. V. Maksimova I. L. Zimnyakov D. A. Kon I. L. Mavlutov A. H. Mishin A. A. (1997). Light propagation in tissues with controlled optical properties. J. Biomed. Opt. 2, 401–417. doi: 10.1117/12.281502, PMID: 23014964

[ref78] Vearrier D. Grundmann O. (2021). Clinical pharmacology, toxicity, and abuse potential of opioids. J. Clin. Pharmacol. 61 Suppl 2, S70–S88. doi: 10.1002/jcph.1923, PMID: 34396552

[ref79] Wang Y. Jiang H. Wang L. Gan H. Xiao X. Huang L.. (2023). Luteolin ameliorates loperamide-induced functional constipation in mice. Braz. J. Med. Biol. Res. 56:e12466. doi: 10.1590/1414-431X2023e12466 36722660 PMC9883005

[ref80] Wood J. D. (1987). “Physiology of the enteric nervous system” in Physiology of the gastrointestinal tract. ed. Johnson L. R. (New York: Raven), 67–110.

[ref81] Yang B. Treweek J. B. Kulkarni R. P. Deverman B. E. Chen C. K. Lubeck E.. (2014). Single-cell phenotyping within transparent intact tissue through whole-body clearing. Cells 158, 945–958. doi: 10.1016/j.cell.2014.07.017, PMID: 25088144 PMC4153367

[ref82] Yuan P. Q. Bellier J. P. Li T. Kwaan M. R. Kimura H. Taché Y. (2021a). Intrinsic cholinergic innervation in the human sigmoid colon revealed using CLARITY, three-dimensional (3D) imaging, and a novel anti-human peripheral choline acetyltransferase (hpChAT) antiserum. Neurogastroenterol. Motil. 33:e14030. doi: 10.1111/nmo.14189 33174295 PMC8126258

[ref83] Yuan P. Q. Kimura H. Million M. Bellier J. P. Wang L. Ohning G. V.. (2005). Central vagal stimulation activates enteric cholinergic neurons in the stomach and VIP neurons in the duodenum in conscious rats. Peptides 26, 653–664. doi: 10.1016/j.peptides.2004.11.015, PMID: 15752581 PMC8082755

[ref84] Yuan P. Q. Wu S. V. Stengel A. Sato K. Taché Y. (2021b). Activation of CRF1 receptors expressed in brainstem autonomic nuclei stimulates colonic enteric neurons and secreto-motor function in male rats. Neurogastroenterol. Motil. 33:e14189. doi: 10.1111/nmo.14189, PMID: 34215021

[ref85] Ziegler A. Gonzalez L. Blikslager A. (2016). Large animal models: the key to translational discovery in digestive disease research. Cell. Mol. Gastroenterol. Hepatol. 2, 716–724. doi: 10.1016/j.jcmgh.2016.09.003, PMID: 28090566 PMC5235339

